# Triphenyltin recognition by primary structures of effector proteins and the protein network of *Bacillus thuringiensis* during the triphenyltin degradation process

**DOI:** 10.1038/s41598-017-04014-y

**Published:** 2017-06-23

**Authors:** Linlin Wang, Jinshao Ye, Huase Ou, Huaming Qin, Yan Long, Jing Ke

**Affiliations:** 1 School of Environment, Jinan University, Guangzhou, 510632 Guangdong China; 2 Joint Genome Institute, Lawrence Berkeley National Laboratory, Walnut Creek, 94598 CA USA

## Abstract

Herein, triphenyltin (TPT) biodegradation efficiency and its transformation pathway have been elucidated. To better understand the molecular mechanism of TPT degradation, the interactions between amino acids, primary structures, and quaternary conformations of effector proteins and TPT were studied. The results verified that TPT recognition and binding depended on amino acid sequences but not on secondary, tertiary or quaternary protein structure. During this process, TPT could change the molecular weight and isoelectric point of effector proteins, induce their methylation or demethylation, and alter their conformation. The effector proteins, alkyl hydroperoxide reductase and acetyl-CoA acetyltransferase, recognizing TPT were crucial to TPT degradation. Electron transfer flavoprotein subunit alpha, phosphoenolpyruvate carboxykinase, aconitate hydratase, branched-chain alpha-keto acid dehydrogenase E1 component, biotin carboxylase and superoxide dismutase were related to energy and carbon metabolism, which was consistent with the results *in vivo*. The current findings develop a new approach for investigating the interactions between proteins and target compounds.

## Introduction

Organotins have been widely used as antifouling biocides, polyvinyl chloride stabilizers, catalysts and agricultural pesticides since the 1960s. Their current applications as organic light-emitting diodes, antitumor agents, fluorescent bioimaging and optical nonlinear materials have led to increased levels of pollution^1, 2^. The widespread use of triphenyltin (TPT), a representative organotin with endocrine disrupting effects^3^, has led to global environmental contamination.

Biodegradation is a primary approach used to reduce TPT and its derivatives in a natural environment without exposure to ultraviolet radiation. Regarding the effective microbes for TPT degradation, *Bacillus thuringiensis* can effectively cleave the carbon-tin bonds of organotins and degrade organotins^4, 5^. This spore-forming species was selected in the current study due to its use as a commonly used biopesticide without negative impacts on humans, wildlife, and pollinators^6^; its TPT degradation ability; and its resistance to a variety of stresses, including pollutant toxicity. Metabolite analysis has revealed that TPT is degraded by *B. thuringiensis* through the successive dephenylation pathway, producing diphenyltin, monophenyltin and tin^4^. This process has been associated with the metabolism of ions, carbohydrates and organic acids^7^ and is regulated by cellular protein networks. The elucidation of the metabolic mechanism related to the interactions between TPT degradation and cellular metabolism would undoubtedly present a novel technology for organotin biodegradation through metabolic regulation.

Proteomics focusing on identifying whole cellular proteins, quantitatively detecting the changes of protein expression and revealing the interactions among proteins would be an attractive approach to reveal the cellular metabolism of effective microbes during the pollutant biodegradation process. However, high-throughput approaches and traditional methods used to identify individually target proteins all rely on the spatial conformations of target proteins. For example, Harada *et al*. reported that a tin atom contacted with a sulfur atom of Cys285 in the peroxisome proliferator-activated receptor and interacted with helix 12 of the receptor ligand-binding domain^8^.

Each protein is a three-dimensional arrangement of molecules and consists of a unique primary structure, which determines its secondary, tertiary and quaternary conformations. Protein motifs have also been identified by their amino acid (AA) sequence. Therefore, it can be deduced that effector proteins can recognize and bind target pollutants by relying only on their AA sequences rather than their spatial conformations. If this hypothesis is correct, the primary structures of effector proteins may recognize the target pollutants and form complexes. Two-dimensional gel electrophoresis (2DE) is one of the proteomic analysis approaches used to separate proteins according to their isoelectric point and molecular weight. In this process, any disulfide bonds in the proteins will have been broken. Proteins that can recognize the target compounds and form complexes with them relying on their primary structures would alter their positions in the gel compared with the control gel because of the change in molecular weight or moving speed. Therefore, 2DE could innovatively be used to determine whether pollutants can be recognized by protein primary structures and even reveal the binding sites of effector proteins.

The findings could give insight into functional verification of target proteins, and the selection of protein ligands, target proteins and effective microbes. For example, effective microbes for pollutant degradation could be selected based on pollutant structures and AA sequences presented in protein databases. If the proteome of some strains contains AA sequences homologous to those that have been proven to transform the target pollutants, it means that those strains might be the potential effective microbes.

To certify the above hypothesis, 2DE and isobaric tags for relative and absolute quantification (iTRAQ) technology were used to investigate TPT recognition, transport and degradation by protein primary structures or spatial conformations. A new approach was developed to study direct interactions between effector proteins and target pollutants and was conducted by separately adding TPT to silver nitrate staining solution, low melting point agarose and denatured protein solution during the 2DE process.

## Results and Discussion

TPT can be effectively degraded by *B. thuringiensis*, successively producing diphenyltin and monophenyltin. The gas chromatograph-mass spectra of these products are shown in the supporting information (Supplementary Figure S1). To reveal the molecular mechanism related to the interactions between TPT and its effector proteins during the degradation process, proteomic analysis was performed in the current study. In 2DE analysis, the secondary, tertiary and quaternary structures of proteins were reduced by the compounds in the lysis and equilibration buffers. Among them, urea and thiourea open peptide chains by breaking hydrogen bonds and other secondary bonds, dithiothreitol destroys disulfide bonds among or in protein subunits, iodoacetamide blocks the formation of disulfide bonds, sodium dodecyl sulfate and 3-[(3-cholamidopropyl)dimethylammonio]propanesulfonate damage hydrophobic interactions and bonds among or in proteins, phenylmethylsulfonyl fluoride represses serine hydrolase and cysteine hydrolase, and nucleases repress DNAzyme and RNase.

**Figure 1:**
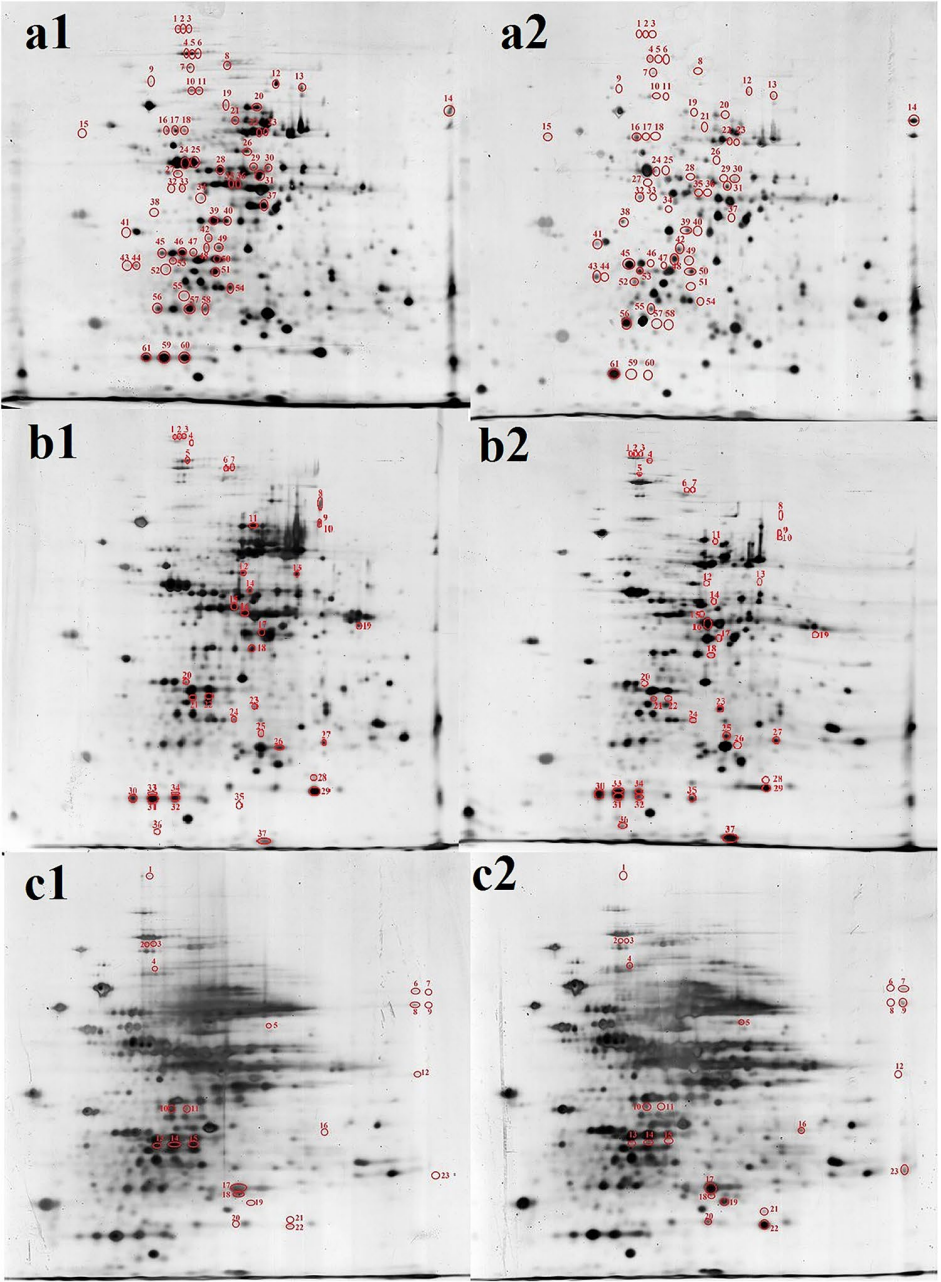
Interaction between TPT and protein primary structures in different conditions. (**a**) silver staining process; (**b**) TPT was added in low melting point agarose gel; (**c**) TPT and denatured proteins mixed in lysis buffer. 1: control samples; 2: experimental samples.

Recognition is the initial step for substrate transformation through catalysis by effector proteins. It is thus crucial to study which proteins recognize and bind to TPT. To this end, the interaction between protein primary structures and TPT in different conditions was investigated. Silver nitrate staining solution with 1 mg L^−1^ TPT was used to stain the effector proteins in polyacrylamide gels after electrophoresis. The staining intensities and positions of the staining spots could reflect whether TPT contacted proteins in gels directly, possibly changing their conformations by folding or binding (Fig. 1a). TPT at a concentration of 1 mg L^−1^ in low melting point agarose was used to react with proteins in IPG-strip gels during the electrophoresis process (Fig. 1b). The molecular weight of TPT is smaller than proteins, thus it runs faster than proteins during the electrophoresis process. If proteins could recognize TPT and form complexes, the increase in molecular weight of the complexes would slow their electrokinetic speeds, which can then be detected by comparing the spot positions between gels. Alternatively, TPT bound to different AAs may result in protein folding and a subsequent increased migration rate as protein structure is more compact. To determine whether the interaction between protein primary structures and TPT formed stable complexes or unstable intermediates, proteins obtained after cell lysis were denatured in lysis buffer, mixed with TPT and subsequently separated by 2DE (Fig. 1c).

Spots 59–61 in Fig. 1a and spots 30–34 in Fig. 1b correspond to alkyl hydroperoxide reductase C22 with different modifications. Among them, the staining intensities of spots 59 and 60 in Fig. 1a and spots 31 and 32 in Fig. 1b are lower, whereas those of spot 61 in Fig. 1a and spots 33–35 in Fig. 1b are higher. The separation of spots 33 and 34 from spots 31 and 32 (Fig. 1b) confirms that TPT can bind to various AAs in one protein, resulting in a more compact structure and an increase in migration rate^9^. This finding also illustrated that TPT recognition by effector proteins was a rapid process. The light color of spots 59 and 60 in Fig. 1a verifies that protein folding led to a decrease in binding sites for Ag^+^. Its up-regulated expression in viable cells after TPT degradation further demonstrated alkyl hydroperoxide reductase is an effector protein (Supplementary Table S5) for the catalysis of organic hydroperoxides under TPT stress^10^.

As for zinc-containing putative alcohol dehydrogenase (spot 16, Fig. 1b), TPT could not only be recognized by it, but also induce its methylation and AA sequence change, forming alcohol dehydrogenase.

Spots 6–9 in Fig. 1c reveal that TPT interacted with propionyl-CoA carboxylase and inosine 5′-monophosphate dehydrogenase, changing their isoelectric points and causing their modification. This resulted in the generation of inositol-5-monophosphate dehydrogenase, which is a key enzyme in controlling cellular nucleotide pools and is involved in cell growth and apoptosis^11^. This reason is why new organotins were synthesized by using TPT as the main component to control HIV and some cancer cells^12, 13^. The divergence between the AA sequences of inosine 5′-monophosphate dehydrogenase (isoleucine) and inositol-5-monophosphate dehydrogenase (valine) is one AA. In comparison to isoleucine, valine has one more methylene group, thus suggesting that TPT triggered the demethylation of inosine 5′-monophosphate dehydrogenase.

The change of spot 14 (from propionyl-CoA carboxylase to propionyl-CoA carboxylase beta chain) in Fig. 1a means the effect of TPT on the protein varied in different conditions. TPT resulted in the molecular modification and AA sequence change of propionyl-CoA carboxylase in the silver staining process but only altered the electrical property of propionyl-CoA carboxylase in the lysate solution. In addition, ribosome-associated factor Y involved in protein synthesis showed darker staining in Fig. 1c but lighter staining in Fig. 1b. It is deduced that the tin atom in TPT bound to some AAs of ribosome-associated factor Y and provided more binding sites for Ag^+^ in the solution condition because Ag^+^ can bind to benzene rings in TPT^14^. Tin atoms and benzene rings in TPT bound to different AAs of the protein during electrophoresis, resulting in protein folding and a subsequent decrease in Ag^+^ binding sites.

In the current experiments, the direct interaction between protein primary structures and TPT was analyzed. Insight into the relationship between TPT and effector proteins is supported by the new approach examining the direct interaction between proteins and TPT. Protein spots with different staining intensities in Fig. 1 demonstrate that the protein primary structures can recognize and interact with TPT rapidly. The reactions include TPT binding, TPT-protein complex formation, peptide chain folding, and protein molecular weight alteration. The secondary, tertiary and quaternary structures of proteins are dispensable to target substrate recognition and binding. For TPT, it could not only be recognized by protein primary structures, but it can also alter the molecular weight, isoelectric point, primary structure and even amino acid residues of proteins. The decrease in the staining intensities of some proteins in 2DE gels resulted from the combination of tin atom and benzene rings in TPT with AAs in the protein leading to protein folding and reducing the AAs for Ag^+^ binding, whereas the increase in the staining intensities of these proteins was due to the combination of only tin atoms of TPT to proteins. Benzene rings in TPT could also be bound by Ag^+^.

**Figure 2:**
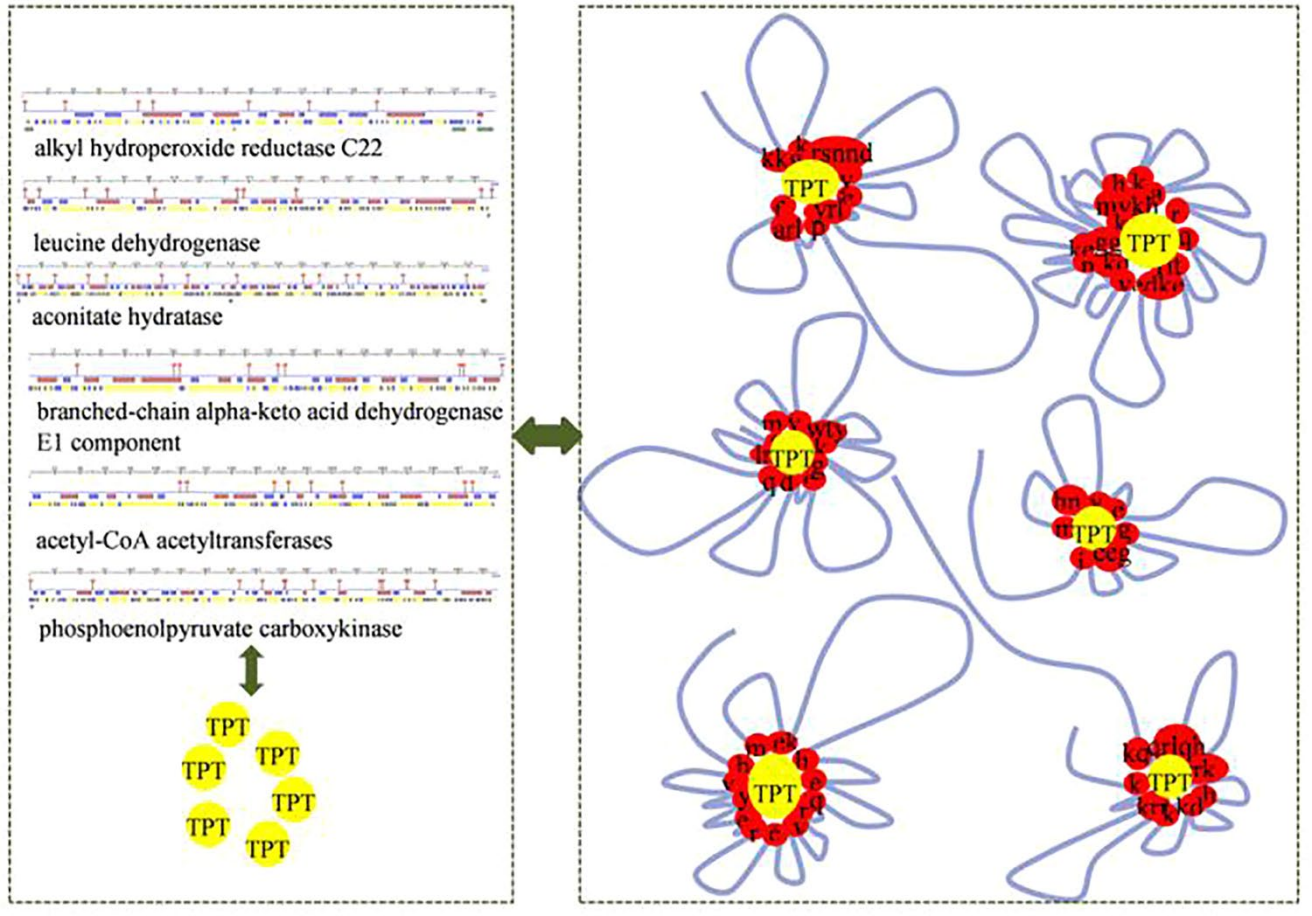
TPT recognition and combination by effector proteins in 2DE experiments.

During the 2DE process, TPT could fold peptide chains and increase protein migration speed in gels by using both tin atom and benzene rings to interact with AAs in the protein (Fig. 2). The interaction between TPT and proteins observed in 2DE experiments involving protein conformation change and TPT binding was crucial to TPT recognition, transport and degradation.

To reveal the interaction between pollutants and proteins, and further determine the active sites of proteins, the binding between pollutants and AAs was investigated. The studied AAs in the current experiment included alanine (ALA), cysteine (CYS), aspartic acid (ASP), glutamic acid (GLU), phenylalanine (PHE), glycine (GLY), histidine (HIS), isoleucine (ILE), lysine (LYS), leucine (LEU), methionine (MET), asparagine (ASN), proline (PRO), glutamine (GLN), arginine (ARG), serine (SER), threonine (THR), valine (VAL), tryptophan (TRP) and tyrosine (TYR). The background values of TPT with AAs or ninhydrin were deducted in all samples. In addition, LC–MS/MS (TripleTOF™ 5600 + ; Agilent Poroshell 120 EC–C_18_ column, 100 × 4.6 mm, 2.7 μm) was used to analyze the TPT medium after boiling treatment and compare it with the same medium without boiling treatment to identify whether the boiling process would result in the hydrolysis of TPT. The results showed that only TPT (Ph_3_Sn^+^) was detected in the media regardless if boiling treatment was used (Supplementary Figure S2). Therefore, the differences in the OD_570nm_ values were due to the interaction between TPT and AAs.

**Figure 3:**
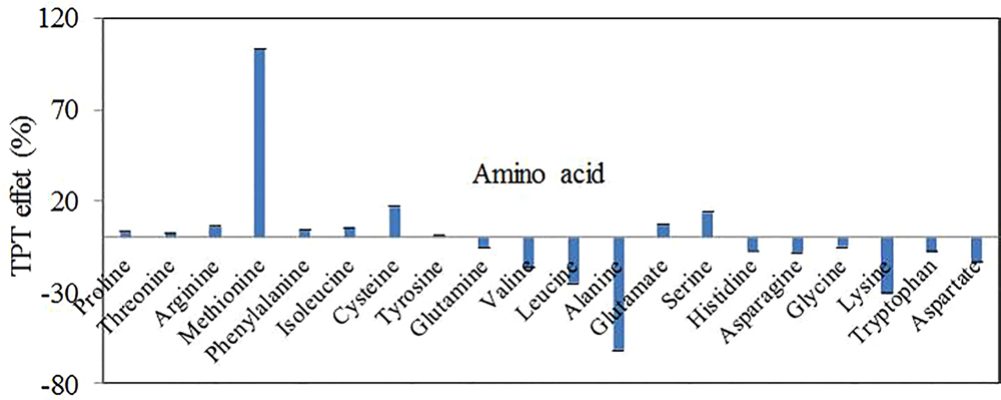
Interaction between TPT and AAs.

It is notable that TPT can change the OD_570nm_ values of all the AAs interacting with ninhydrin. The value changes of ALA, CYS, ASP, GLU, PHE, GLY, HIS, ILE, LYS, LEU, MET, ASN, PRO, GLN, ARG, SER, THR, VAL, TRP and TYR were –61.6, 16.9, –13.8, 7.4, 4.8, –5.4, –7.2, 5.5, –30.7, –25.1, 103.9, –8.1, 3.4, –5.5, 6.3, 14.0, 2.8, –16.9, –7.7 and 1.2%, respectively (Fig. 3). To verify whether AA composition influences the interaction between proteins and TPT, the percentages of each AA in all proteins detected in 2DE experiments and other unrelated proteins in *B. thuringiensis* were calculated and shown in the heatmaps. Supplementary Figure S3 reveals that there is no obvious difference among effector proteins with primary structures that could react with TPT (Supplementary Figure S3a) and that there is also no significant difference among unrelated proteins (Supplementary Figure S3b). However, the AA composition did exhibit a significant difference between effector proteins and those that did not react with TPT (Supplementary Figure S3c). Effector proteins have more ALA, PHE, SER, HIS, CYS and PRO, but no ASP, TRP and ILE, whereas the unrelated ones have more LEU, ILE, VAL, ALA, GLY, LYS and GLU. However, the score (1992; Supplementary Table S1, summary of AA weight × AA percentage) of effector proteins is only slightly higher than that (1807) of unrelated proteins, suggesting that the TPT rapid recognition sites of proteins comprised special AA sequences and patterns instead of the simple AA composition in proteins. The AA weights in Supplementary Table S1 were given by the absolute values of AAs’ OD_570nm_ value changes because these values reflected that TPT could interact with AAs regardless of whether the OD_570nm_ values increased or decreased.

Although it was deduced that tin atoms of TPT react with free sulfhydryl groups in proteins^15^ and that TPT induces conformational changes of albumin by hydrophobic forces and forms TPT-albumin complexes^16^, those conclusions were inferred through experiments using active cells but not through direct evidence of interactions between proteins and TPT. Based on functional analysis in the STRING and NCBI databases, most of the proteins interacting with TPT were involved in carbon source, amino acid, fatty acid and energy metabolism; redox processes; and protein synthesis (Supplementary Tables 2–4. These results conformed to those of iTRAQ (Supplementary Tables S5–6), illustrating that proteins with primary structures involved in TPT recognition and binding were related to cell metabolism and TPT degradation.

The analysis was subsequently restricted to protein spots with similar alterations in staining within 2DE experiments. Biomarkers with light staining included alkyl hydroperoxide reductase C22, electron transfer flavoprotein subunit alpha, superoxide dismutase (SOD), aconitate hydratase, elongation factor G2, phosphoenolpyruvate carboxykinase, biotin carboxylase 2, branched-chain alpha-keto acid dehydrogenase E1 component and leucine dehydrogenase.

**Figure 4:**
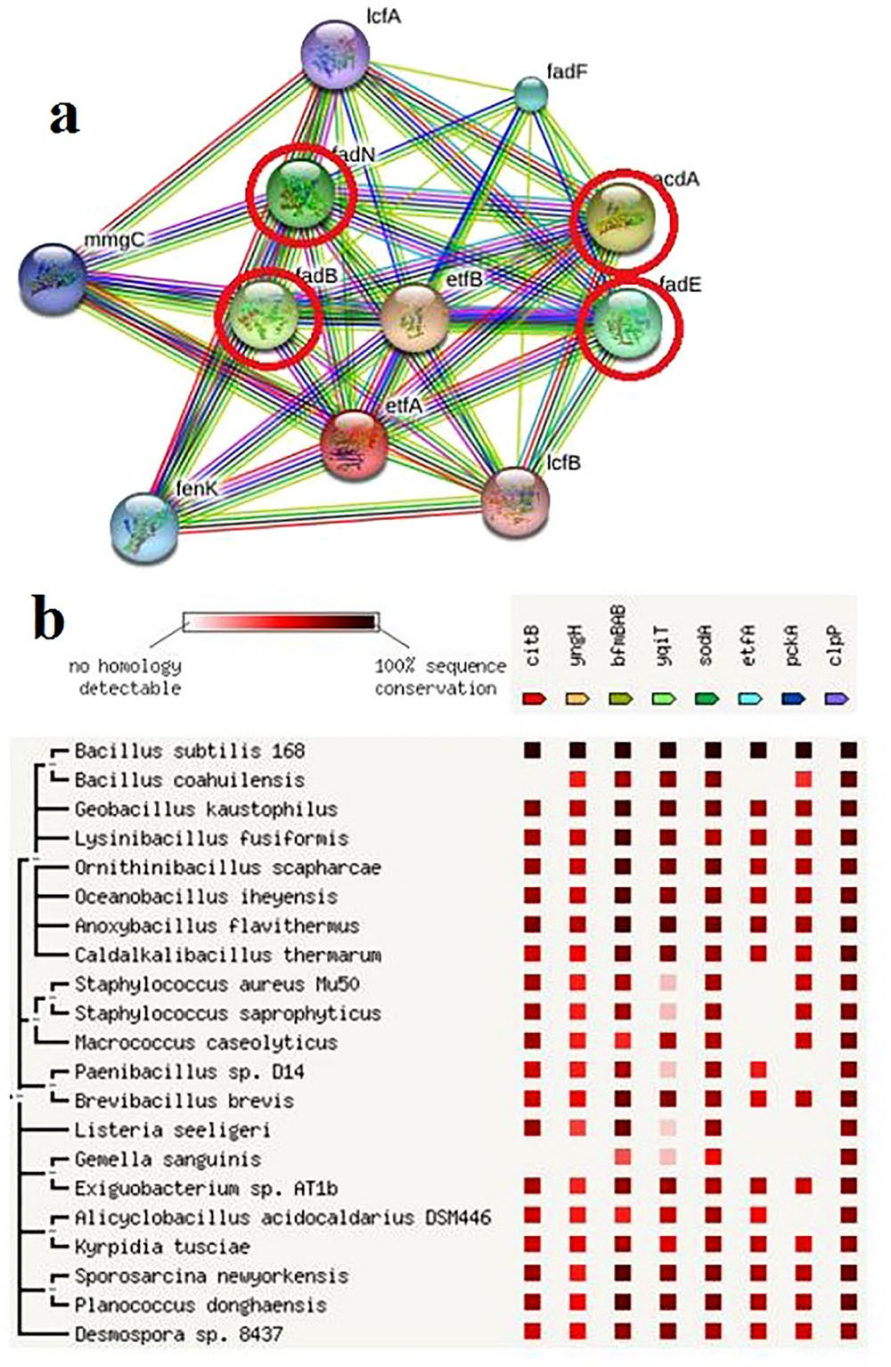
Analysis of effector proteins with same expression trends in different experiments. (**a**) protein network of electron transfer flavoprotein subunit alpha (medium confidence: 0.400); (**b**) homology analysis of different species.

Electron transfer flavoprotein subunit alpha serves as a specific electron acceptor and is involved in the redox process and was up-regulated during TPT degradation (Supplementary Table S5). Its neighborhood proteins, acyl-CoA dehydrogenases and enoyl-CoA hydratases involved in long-chain fatty acid degradation, were also up-regulated (Fig. 4a). These findings explained why TPT suppresses fatty acid synthesis^4^.

Phosphoenolpyruvate carboxykinase stimulates gluconeogenesis by catalyzing the conversion of oxaloacetate to phosphoenolpyruvate, a rate-limiting step in the metabolic pathway producing glucose^17^. Its up-regulation (Supplementary Tables S5 and 6), enhancing carbon metabolism, helps to explain why TPT promotes cell growth, not by acting as a nutrient but by binding to phosphoenolpyruvate carboxykinase to enhance its activity.

**Figure 5:**
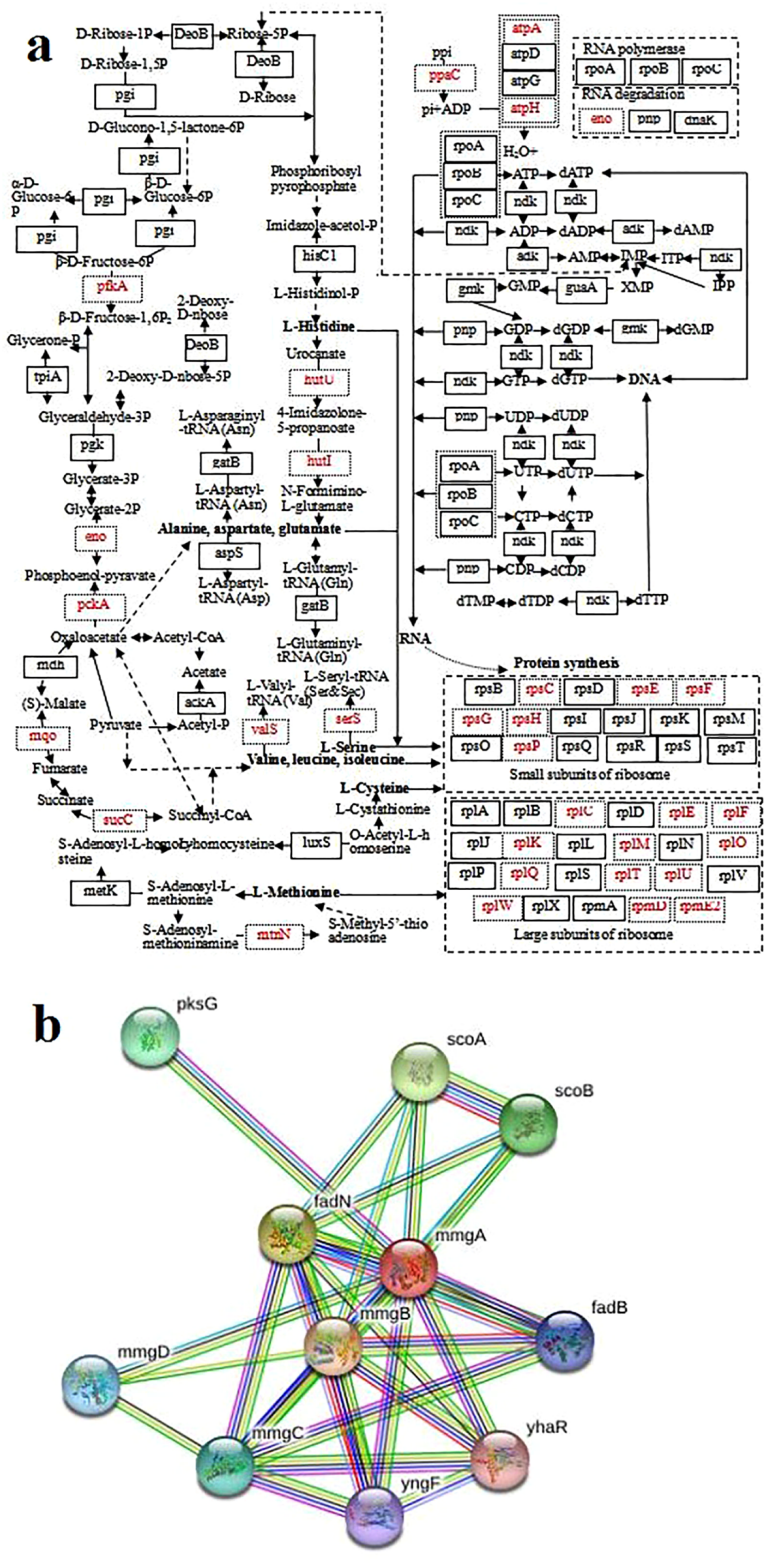
Analysis of the differentially expressed proteins detected by iTRAQ. (**a**) metabolic network regulated by up- and down-regulated synthesis proteins (genes with red dotted line: up-regulation); (**b**) protein network of acetyl-CoA acetyltransferase (medium confidence: 0.400).

Aconitate hydratase is involved in the TCA cycle and glycolysis and showed up-regulation under pollutant stress^18^, which was consistent with its elevated expression in the current experiment (Supplementary Table S5). Branched-chain alpha-keto acid dehydrogenase E1 component is also involved in carbon metabolism. Levels of biotin carboxylase, a subunit of acetyl-CoA carboxylase involved in both fatty acid and pyruvate biosynthesis, are augmented in iTRAQ results (Supplementary Table S5). A previous study^4^ suggests that the limitation of fatty acid synthesis by TPT signifies that biotin carboxylase is associated with gluconeogenesis (Fig. 5b).

SODs have Cu/Zn, Ni, Mn and Fe-binding sites that are used to bind pollutants^19^ and can destroy superoxide anion radicals that are toxic to biological systems. The interaction of SODs with TPT (Supplementary Table S5) implies the improved recovery of cells under TPT stress.

The reduction of leucine dehydrogenase levels (Supplementary Table S5) is associated with the bacterial catabolism of branched-chain L-amino acids and spore germination and occurs in concert with the attenuation of cell mass^4^. It thus serves as a resistant protein to TPT and is depressed or degraded during the TPT degradation process. Therefore, it could be used as a biomarker to reflect environmental pollution.

In terms of the darker-stained proteins, the ATP-dependent Clp protease proteolytic subunit that cleaves some misfolded proteins was an effector protein for TPT degradation. Exposure to TPT can trigger the misfolding of some proteins, although *B. thuringiensis* can use TPT as a carbon nutrient. Therefore, the ATP-dependent Clp protease proteolytic subunit is required for correct protein folding, which was consistent with its up-regulated expression in the iTRAQ results (Supplementary Tables S5 and 6).

Proteins with primary structures that recognize and interact with TPT directly included electron transfer flavoprotein subunit alpha, phosphoenolpyruvate carboxykinase, aconitate hydratase, branched-chain alpha-keto acid dehydrogenase E1 component biotin carboxylase and SOD, TPT degradation proteins such as alkyl hydroperoxide reductase C22, and TPT-resistant proteins such as leucine dehydrogenase. The correlation of these effector proteins to TPT degradation, protein synthesis and cellular metabolism illustrate that they can selectively recognize TPT. Owing to this property of effector proteins, one can deduce that organisms containing the same genes or proteins will exhibit the same or similar responses to TPT. The effective biosorption, transport and degradation of TPT by *Brevibacillus brevis* ^20^ verify the above deduction (Fig. 4b). This finding confirms that the interaction between protein primary structures and pollutants can be an innovative approach to select effective organisms and analyze the physicochemical activities of organisms in the natural environment under the stress of target pollutants.

The proteome response and protein networks in cells related to TPT degradation were revealed by iTRAQ. Fig. 5a shows that TPT degradation tended to trigger the synthesis of proteins associated with pyruvate, amino acid and energy metabolism, glycolysis, the pentose phosphate pathway, the citrate cycle, ribosome metabolism and protein synthesis. The above mentioned processes, especially ribosome metabolism and protein synthesis, were required for various physicochemical activities, rather than merely for TPT degradation. The up regulation of atpA, atpH and ppaC stimulated energy metabolism for protein, AA, and nucleic acid catabolism; carbohydrate catabolism; and other kinds of cellular metabolisms. The up-regulation of pfkA, eno, pckA, mqo and sucC regulates glycolysis, the pentose phosphate pathway, and the citrate cycle as well as protects cells from oxidative stress and DNA damage^21^.

The up-regulation of hutU, hutI, valS and serS enhances the synthesis of GLU and the complex of tRNA with VAL and SER. These findings can be explained by the preference to form GLU-, VAL- and SER-containing proteins when degrading TPT. For example, acetyl-CoA acetyltransferases (Supplementary Table S5), involved in ethylbenzene, benzoate and geraniol degradation (http://www.genome.jp/kegg-bin/show_pathway? ko00642; http://www.genome.jp/kegg-bin/show_pathway?ko00362; http://www.genome.jp/kegg-bin/show_pathway?ko00281), contain a great number of these AAs and show high expression (5.4- and 2.0-fold increased). Furthermore, acetyl-CoA acetyltransferase indirectly promotes pyruvate metabolism and the citrate cycle. The high expression of its surrounding proteins, including mmgB, mmgD and enoyl-CoA hydratases, as presented in Fig. 5b, further verifies the above metabolism. The staining intensity of acetyl-CoA acetyltransferase shown in Fig. 1 is lower, verifying that acetyl-CoA acetyltransferase can interact with TPT.

In summary, the TPT degradation process tends to trigger differential expression of proteins associated with protein, amino acid, carbon and energy syntheses, which was consistent with the results of 2DE experiments.

**Figure 6:**
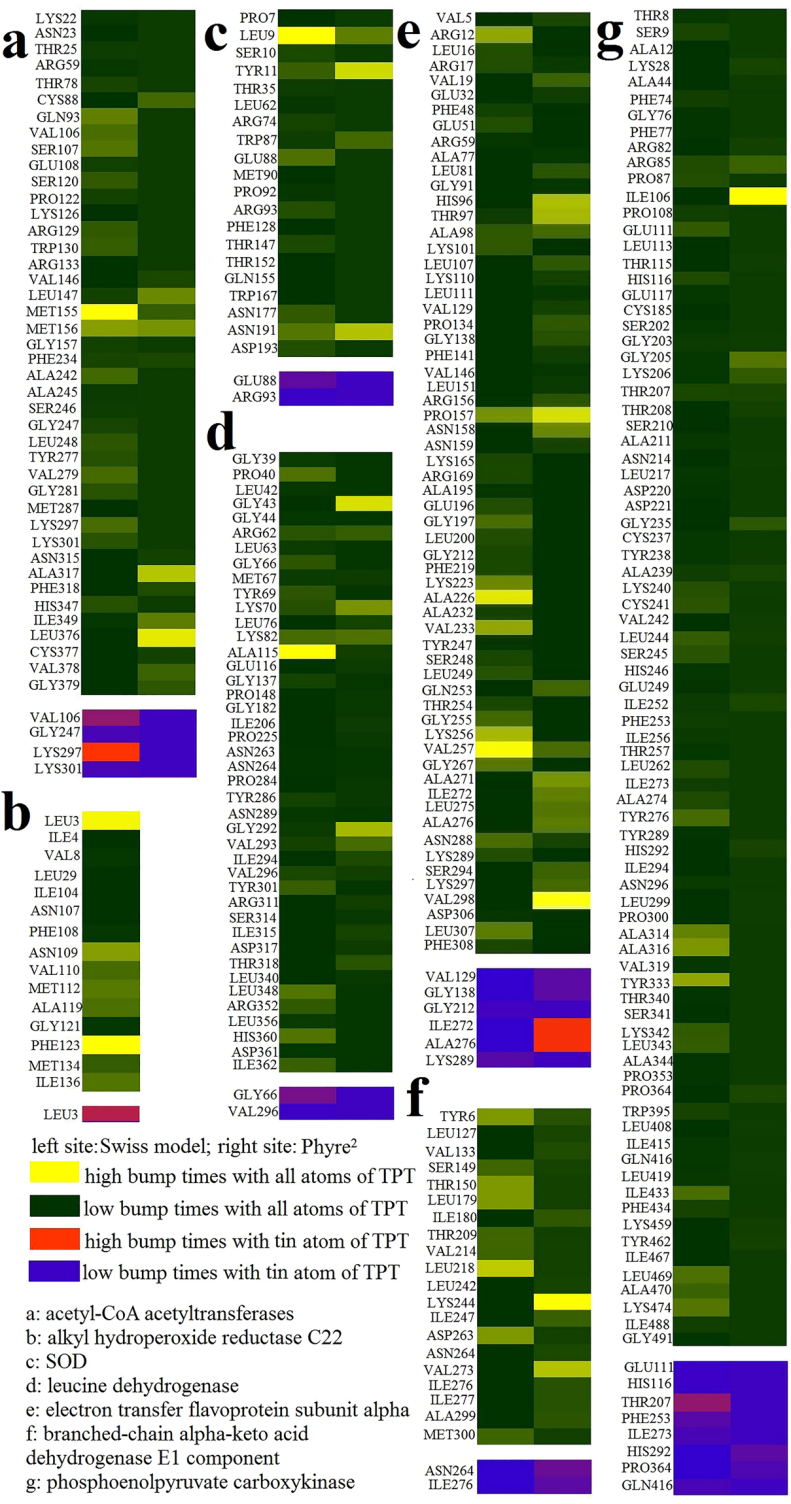
Bump times between proteins and TPT.

Furthermore, some effector proteins (acetyl-CoA acetyltransferase, alkyl hydroperoxide reductase C22, SOD, leucine dehydrogenase, electron transfer flavoprotein subunit alpha, branched-chain alpha-keto acid dehydrogenase E1 component and phosphoenolpyruvate carboxykinase) were selected to analyze their interaction with TPT, and these analyses revealed possible binding sites (Supplementary Figure S4 and Table S7). Protein models were built using two programs, Swiss model (www.swissmodel.expasy.org) and Phyre^2^ (www.sbg.bio.ic.ac.uk), to compare their differences and ensure result reliability. The indexes of receptor and ligand bumps were used to evaluate the interaction between TPT and target proteins. High bump times mean that the possibility of ligand interaction with receptor is high. Figure 6a shows that the bump times between TPT and MET155 of acetyl-CoA acetyltransferase from the Swiss model were high and that bumps also occurred in the Phyre^2^ model. The next AA, MET156, was another highly possible binding site. LYS297 in acetyl-CoA acetyltransferase remained in contact with the tin atom for the most times. Although there was no binding site predicted in alkyl hydroperoxide reductase C22 by Phyre^2^, the Swiss model result (Fig. 6b) shows that LEU3 of alkyl hydroperoxide reductase C22 bumped against TPT frequently and contacted with tin atoms, revealing that LEU3 might be an active binding site. Figure 6c shows that LEU9, TYR11 and ASN191 were the most possible binding sites for SOD. ARG62, LYS70, LYS82 and ALA115 were all highly possible binding sites of leucine dehydrogenase (Fig. 6d). It is likely that PRO157 and VAL257 were the active binding sites of electron transfer flavoprotein subunit alpha (Fig. 6e). In addition, Phyre^2^ predicted that the ILE272 and ALA276 in electron transfer flavoprotein interacted with tin atoms.

## Conclusion

Recognition between proteins and TPT depends on AA sequence and pattern and not on secondary, tertiary or quaternary protein structure. During the recognition and interaction process, TPT may alter the molecular weight and isoelectric point of effector proteins, induce their methylation or demethylation, and alter their conformation. The TPT degradation process tended to trigger differential expression of proteins associated with protein synthesis and amino acid, carbon and energy metabolism. The discoveries of the interactions between TPT and various proteins by using a new approach based on protein primary structure greatly advance the understanding of effective microbe selection and TPT applications.

## Methods

### Strain and chemicals

*B. thuringiensis* GIMCC 1.817, which was selected from the natural environment, was an effective microbe for TPT degradation^3^. Triphenyltin chloride (C_18_H_15_SnCl, CAS No: 639-58-7) was purchased from Sigma–Aldrich (St. Louis, MO, USA). The bacterial culture medium contained 3 g L^−1^ beef extract, 10 g L^−1^ peptone and 5 g L^−1^ NaCl. The concentrations of KH_2_PO_4_, NaCl, NH_4_Cl and MgSO_4_ in the mineral salt medium (MSM) for TPT degradation were 30, 20, 30 and 10 mg L^−1^, respectively.

### TPT biotransformation

*B. thuringiensis* was cultured at 30 °C on a rotary shaker at 130 r min^−1^. After 12 h, the cells were separated at 3500 *g* for 5 min and washed three times with sterile, distilled water. Subsequently, 1 g L^−1^ of cells was inoculated in 20 mL of MSM to degrade 1 mg L^−1^ of TPT in the dark at 130 r min^−1^ for 24 h. After degradation, the cells were separated and washed using pure water for protein extraction, identification and quantification.

### Interaction between TPT and amino acids

Essential AAs at 0.2 g L^−1^, including ALA, CYS, ASP, GLU, PHE, GLY, HIS, ILE, LYS, LEU, MET, ASN, PRO, GLN, ARG, SER, THR, VAL, TRP and TYR, were used to interact with TPT at 1 mg L^−1^ for 30 min via the ninhydrin reaction. Ninhydrin solution (2%, w/v) at 1.5 mL and phosphate buffered solution (pH = 6.7) at 1 mL were added to 2 mL sample solution successively. After heating in boiling water for 18 min, the mixture was promptly cooled down to room temperature in cold water, diluted with water to 30 mL, mixed and stabilized for 15 min, successively. The OD_570nm_ value of each sample was detected by a microplate reader (BioTek, Synergy H1, USA) following the ninhydrin reaction to determine any interactions. The OD_570nm_ value of each AA solution without TPT was set as the control.

### Experimental procedures for 2DE and iTRAQ

Details concerning protein preparation, two-dimensional gel electrophoresis, image generation, in-gel and in-solution digestion, iTRAQ labeling and desalination, and protein identification for 2DE and iTRAQ analysis can be found in the supplementary information file.

### Computational methods

The whole *B. thuringiensis* database was searched, including all subspecies in NCBI. iTRAQ data for further analysis using DAVID (http://david.abcc.ncifcrf.gov; species: *B. thuringiensis*), STRING (http://string-db.org; species: *Bacillus subtilis* 168) and KEGG Pathway (http://www.genome.jp/kegg/pathway.html) were searched in the *B. thuringiensis* database of UNIPROT. The active binding sites of proteins interacting with TPT were analyzed using Discovery Studio software (version 2.5)^22, 23^. The workflow included the steps of preparing ligands, cleaning proteins, finding cavities in receptors, and docking ligands (high quality; dock pose number was up to 100). Protein models were built using the websites of Swiss model and Phyre^2^.

## Electronic supplementary material

Supplementary information

## References

[1] Fan BB, Li HY, Fan WB, Zhang JL, Li RF (2010). Organotin compounds immobilized on mesoporous silicas as heterogeneous catalysts for direct synthesis of dimethyl carbonate from methanol and carbon dioxide. Appl. Catal. A: Gen.

[2] Jiang J (2016). Identification and analysis of triphenyltin chloride with surface enhanced Raman scattering spectroscopy. Chemosphere.

[3] Aydinoglu, S. *et al*. Studies on DNA interaction of organotin(IV) complexes of *meso*-tetra(4-sulfonatophenyl)porphine that show cellular activity. *J. Inorg. Biochem*., doi:10.1016/j.jinorgbio.2016.06.030 (2016). 10.1016/j.jinorgbio.2016.06.030 27393277

[4] Tang LT (2016). Correlation among phenyltins molecular properties, degradation and cellular influences on *Bacillus thuringiensis* in the presence of biosurfactant. Biochem. Eng. J..

[5] Huang J (2014). Triphenyltin biosorption, dephenylation pathway and cellular responses during triphenyltin biodegradation by *Bacillus thuringiensis* and tea saponin. Chem. Eng. J..

[6] Melo ALD, Soccol VT, Soccol CR (2016). *Bacillus thuringiensis*: mechanism of action, resistance, and new applications: a review. Crit. Rev. Biotechnol..

[7] Gao J, Ye JS, Ma JW, Tang LT, Huang J (2014). Biosorption and biodegradation of triphenyltin by *Stenotrophomonas maltophilia* and their influence on cellular metabolism. J. Hazard. Mater..

[8] Shusaku H (2015). Structural basis for PPARγ transactivation by endocrine-disrupting organotin compounds. Sci. Rep.

[9] Czyżnikowska Ż, Bartkowiak W (2011). Physical origins of the stability of aromatic amino acid core ring-polycyclichydrocarbon complexes: a post-Hartree-Fock and density functional study. J. Comput. Chem..

[10] Goto S (2015). Alkyl hydroperoxide reductase enhances the growth of *Leuconostoc mesenteroides* lactic acid bacteria at low temperatures. AMB Express.

[11] Pitaluga AN, Moreira ME, Traub-Csekö YM (2015). A putative role for inosine 5′ monophosphate dehydrogenase (IMPDH) in *Leishmania amazonensis* programmed cell death. Exp. Parasitol..

[12] Liang JW (2014). A series of organotin (IV) complexes based on (E)-3-(3-nitrophenyl) acrylic acid: Syntheses, crystal structures and biological activities. Inorg. Chem. Commun..

[13] Win YF (2015). Synthesis, crystal structures and spectroscopic properties of two new organotin (IV) complexes and their antiproliferative effect against cancerous and non-cancerous cells. C. R. Chimie.

[14] Yan G, Borah AJ, Wang L (2014). Efficient silver-catalyzed direct sulfenylation and selenylation of rich arenes. Org. Biomol. Chem..

[15] Shpakovsky DB (2014). Synthesis, antiradical activity and *in vitro* cytotoxicity of novel organotin complexes based on 2,6-di-tert-butyl-4-mercaptophenol. Dalton. Trans..

[16] Geng B (2014). Investigation on the interaction between endocrine disruptor triphenyltin with human serum albumin. Spectrochim. Acta A Mol. Biomol. Spectrosc..

[17] Stark R (2014). A role for mitochondrial phosphoenolpyruvate carboxykinase (PEPCK-M) in the regulation of hepatic gluconeogenesis. J. Biol. Chem..

[18] Immel F, Renaut J, Masfaraud JF (2012). Physiological response and differential leaf proteome pattern in the European invasive Asteraceae *Solidago canadensis* colonizing a former cokery soil. J. proteomics.

[19] Kim BM (2015). Modulated expression and enzymatic activity of the monogonont rotifer Brachionus koreanus Cu/Zn- and Mn-superoxide dismutase (SOD) in response to environmental biocides. Chemosphere.

[20] Ye JS (2013). Biosorption and biodegradation of triphenyltin by *Brevibacillus brevis*. Bioresour. Technol..

[21] Alam MM, Iqbal S, Naseem I (2015). Ameliorative effect of riboflavin on hyperglycemia, oxidative stress and DNA damage in type-2 diabetic mice: Mechanistic and therapeutic strategies. Arch. Biochem. Biophys..

[22] Liang L (2016). Synthesis, Molecular modeling and biological evaluation of 4-alkoxyquinazoline derivatives as novel inhibitors of VEGFR2. Chem. Pharm. Bull..

[23] Manisha M, Parakh S, Faiza SK, Mrunmai P (2017). Insilico studies on taste receptor gene (Tas2r38) and Tas2r38 protein interaction with ligands PTC and PROP using docking approach. IJARIIT..

