# Supplementary material for: Triphenyltin recognition by primary structures of effector proteins and the protein network of Bacillus thuringiensis during the triphenyltin degradation process
Source: Sci Rep. 2017 Jun 23;7:4133. doi: 10.1038/s41598-017-04014-y (PMC5482883; doi:10.1038/s41598-017-04014-y)
Supplement: Supplementary file 1 — Supplementary information [file 41598_2017_4014_MOESM1_ESM.pdf]

Triphenyltin recognition by primary structures of effector proteins and the protein network of *Bacillus thuringiensis* during the triphenyltin degradation process

Linlin Wang,[1,2] Jinshao Ye,[1,2]* Huase Ou,[1] Huaming Qin,[1] Yan Long,[1] Jing Ke,[2]

[1] *School of Environment, Jinan University, Guangzhou 510632, Guangdong, China*

[2] *Joint Genome Institute, Lawrence Berkeley National Laboratory, Walnut Creek 94598, CA, USA*

*Corresponding author. Tel: +86 13922287937

E-mail: folaye@126.com, jinshaoye@lbl.gov

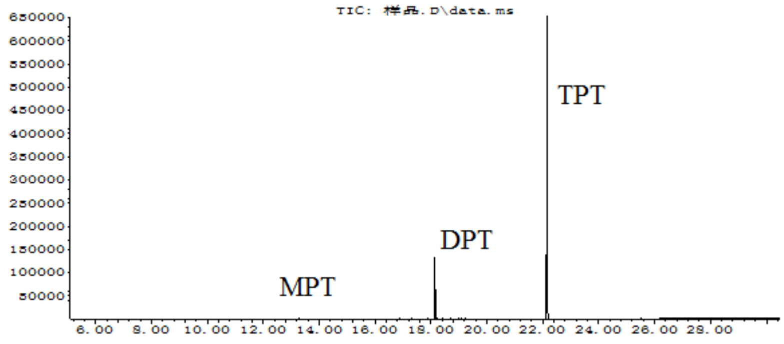

a: GC–MS spectra of TPT and its metabolites after degradation

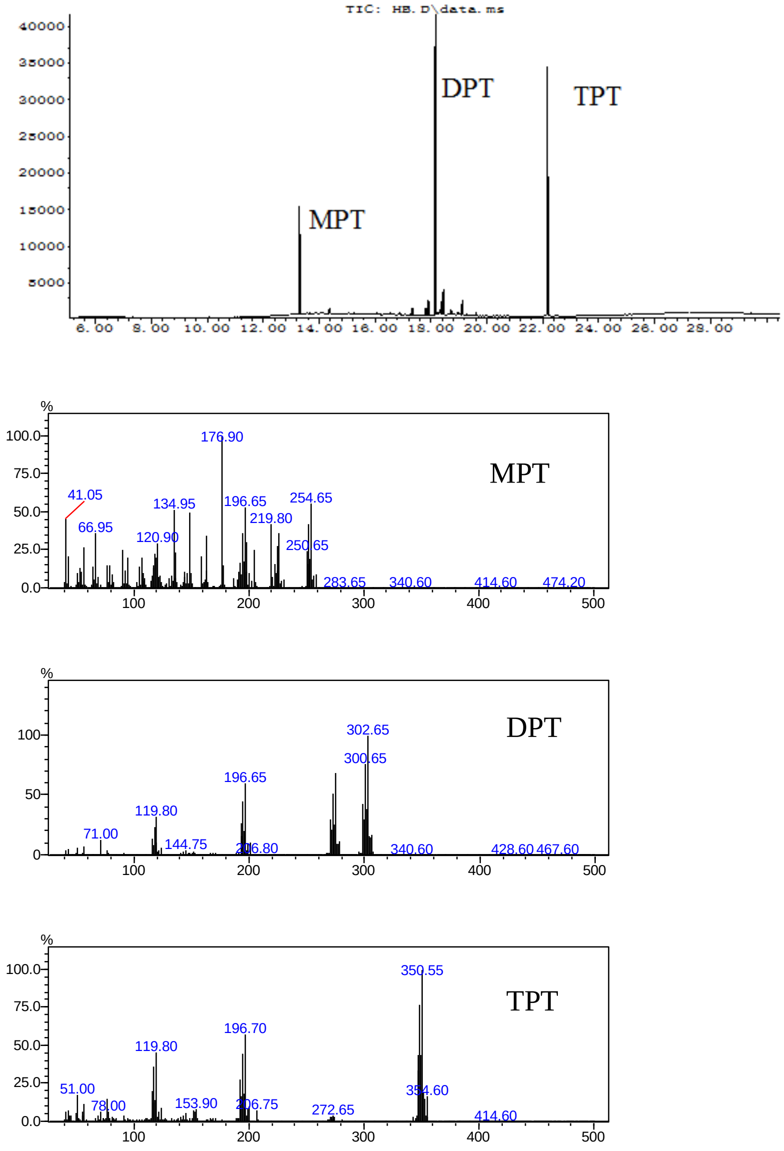

b: GC–MS spectra of TPT, DPT and MPT

Supplementary Figure S1. The GC–MS results of TPT degradation products by *B. thuringiensis*.

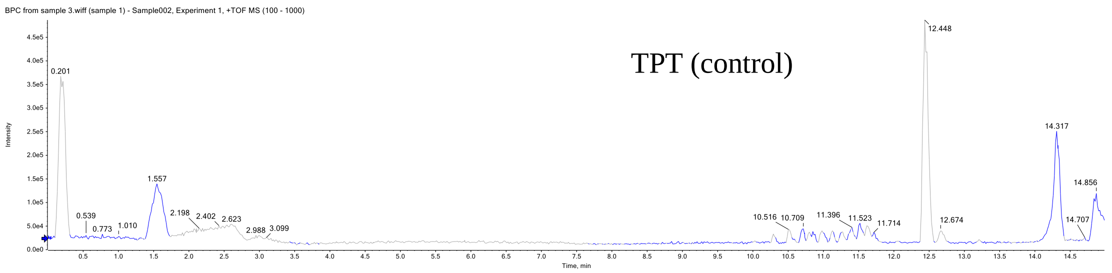

Compound name (library hit)	Formula	Intensity	Expected m/z	Found at m/z	Error (ppm)	Found RT (min)	RT delta (min)
TPT	$C_{18}H_{15}Sn$	2024940	351.0196	351.0199	1.0	12.45	12.45
385.988427329 (*No data for Library Hit Name xcm*)	$C_{18}H_{15}SnCl$	0	385.9884	0.0000	0.0	0.00	0.00
368.02231428 (No Acquired MSMS)	$C_{18}H_{15}SnOH$	32079	368.0223	368.0316	25.3	12.45	12.45
275.99609948 (*No data for Library Hit Name xcm*)	$C_{12}H_{12}Sn$	0	275.9961	0.0000	0.0	0.00	0.00
273.9804494 (*No data for Library Hit Name xcm*)	$C_{12}H_{10}Sn$	0	273.9804	0.0000	0.0	0.00	0.00
199.96479932 (*No data for Library Hit Name xcm*)	C_6H_8Sn	0	199.9648	0.0000	0.0	0.00	0.00
196.9413242 (*No data for Library Hit Name xcm*)	C_6H_5Sn	0	196.9413	0.0000	0.0	0.00	0.00

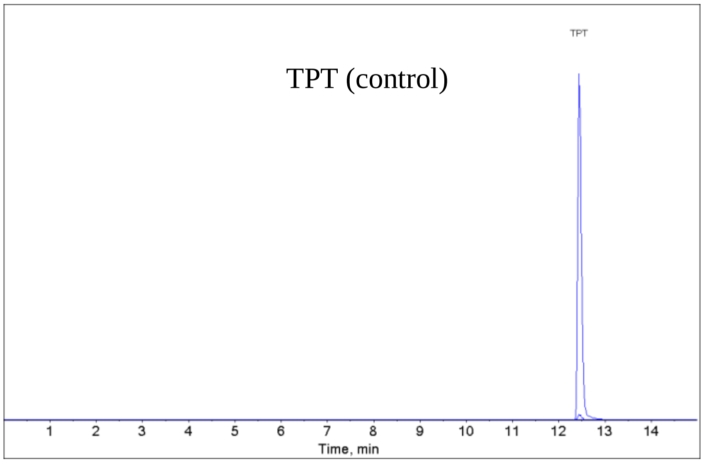

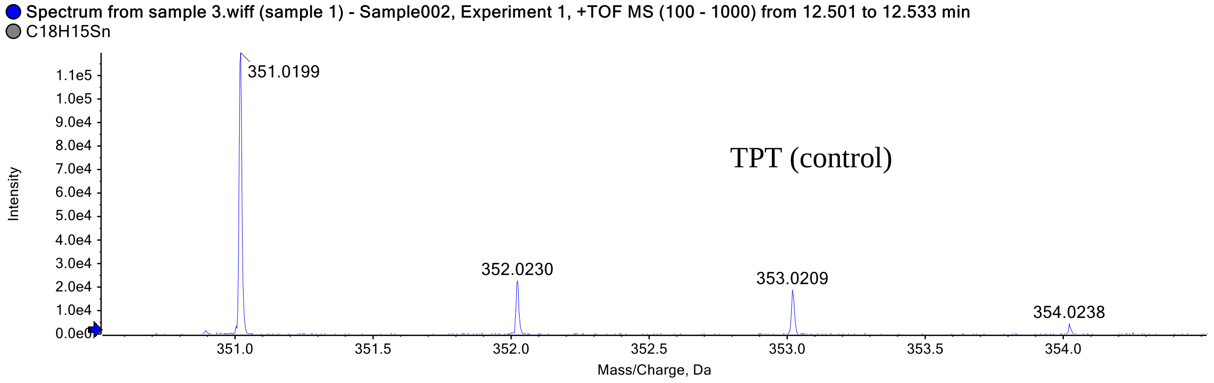

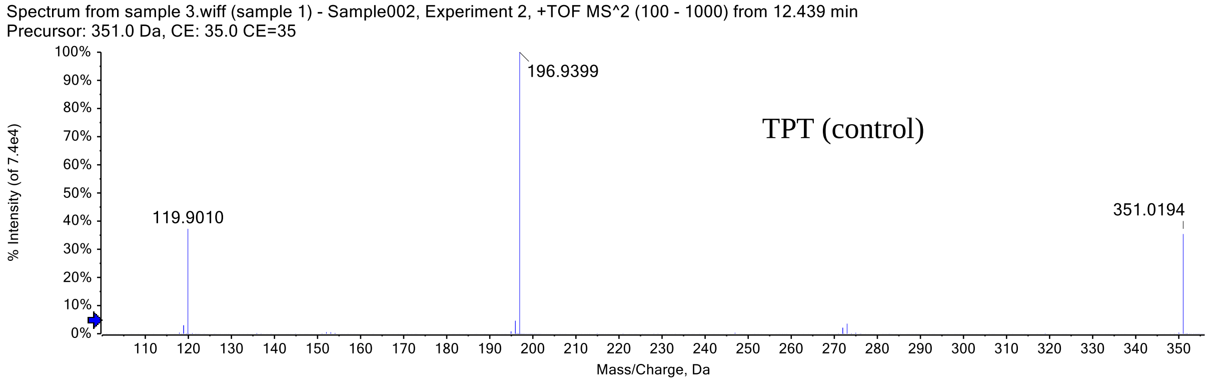

a: LC–MS^2 results of the TPT control sample

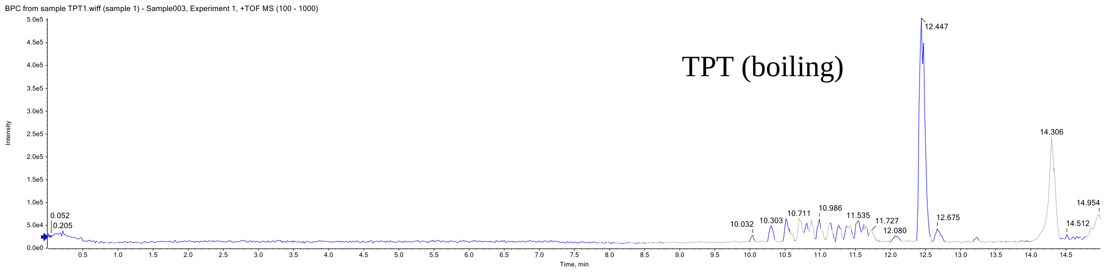

Compound name (library hit)	Formula	Intensity	Expected m/z	Found at m/z	Error (ppm)	Found RT (min)	RT delta (min)
TPT	$C_{18}H_{15}Sn$	2165437	351.0196	351.0200	1.1	12.45	12.45
385.988427329 (*No data for Library Hit Name xcm*)	$C_{18}H_{15}SnCl$	0	385.9884	0.0000	0.0	0.00	0.00
368.02231428 (No Acquired MSMS)	$C_{18}H_{15}SnOH$	29012	368.0223	368.0318	25.7	12.45	12.45

275.99609948 (*No data for Library Hit Name xcm*)	$C_{12}H_{12}Sn$	201	275.9961	0.0000	0.0	0.00	0.00
273.9804494 (*No data for Library Hit Name xcm*)	$C_{12}H_{10}Sn$	0	273.9804	0.0000	0.0	0.00	0.00
199.96479932 (*No data for Library Hit Name xcm*)	C_6H_8Sn	724	199.9648	0.0000	0.0	0.00	0.00
196.9413242 (*No data for Library Hit Name xcm*)	C_6H_5Sn	0	196.9413	0.0000	0.0	0.00	0.00

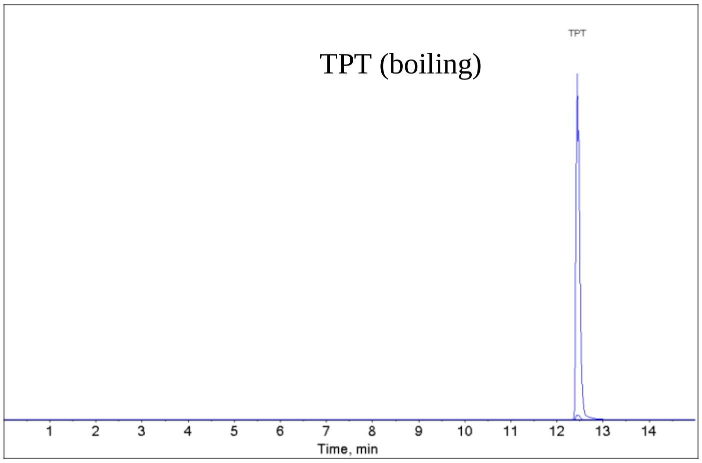

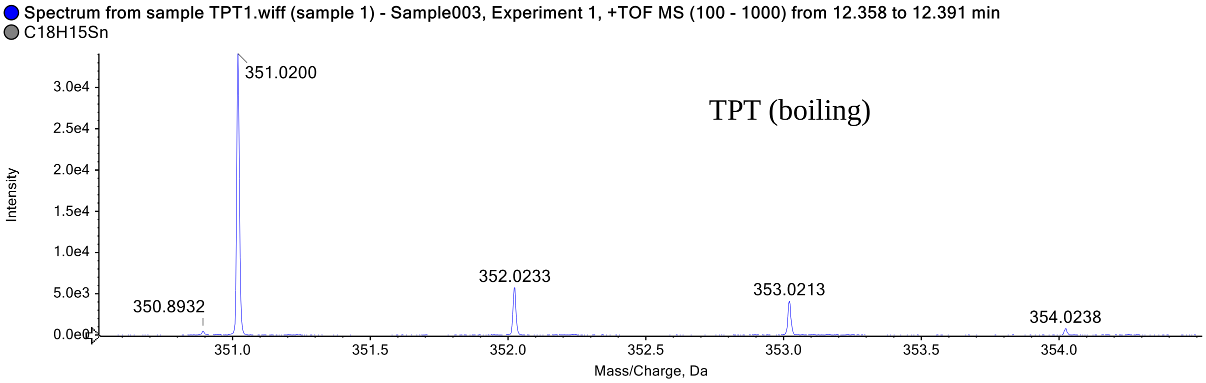

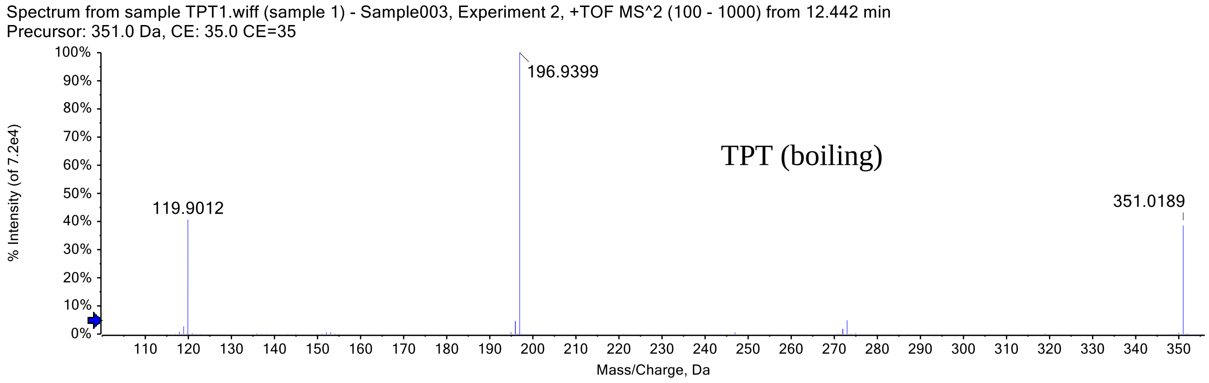

b: LC–MS^2 results of the TPT sample with boiling treatment

Supplementary Figure S2. LC–MS^2 results of the TPT samples with or without boiling treatment.

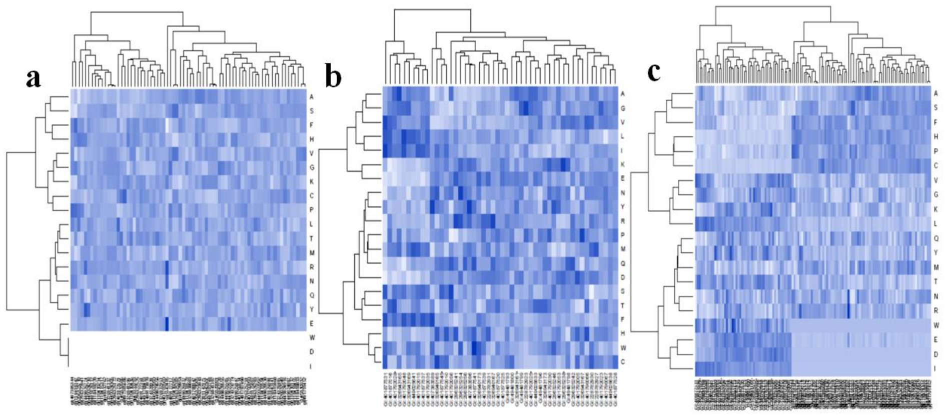

Supplementary Figure S3. The comparison of AA composition among different proteins. a: target proteins identified in 2 DE experiments; b: ineffective proteins; c: target proteins and ineffective proteins.

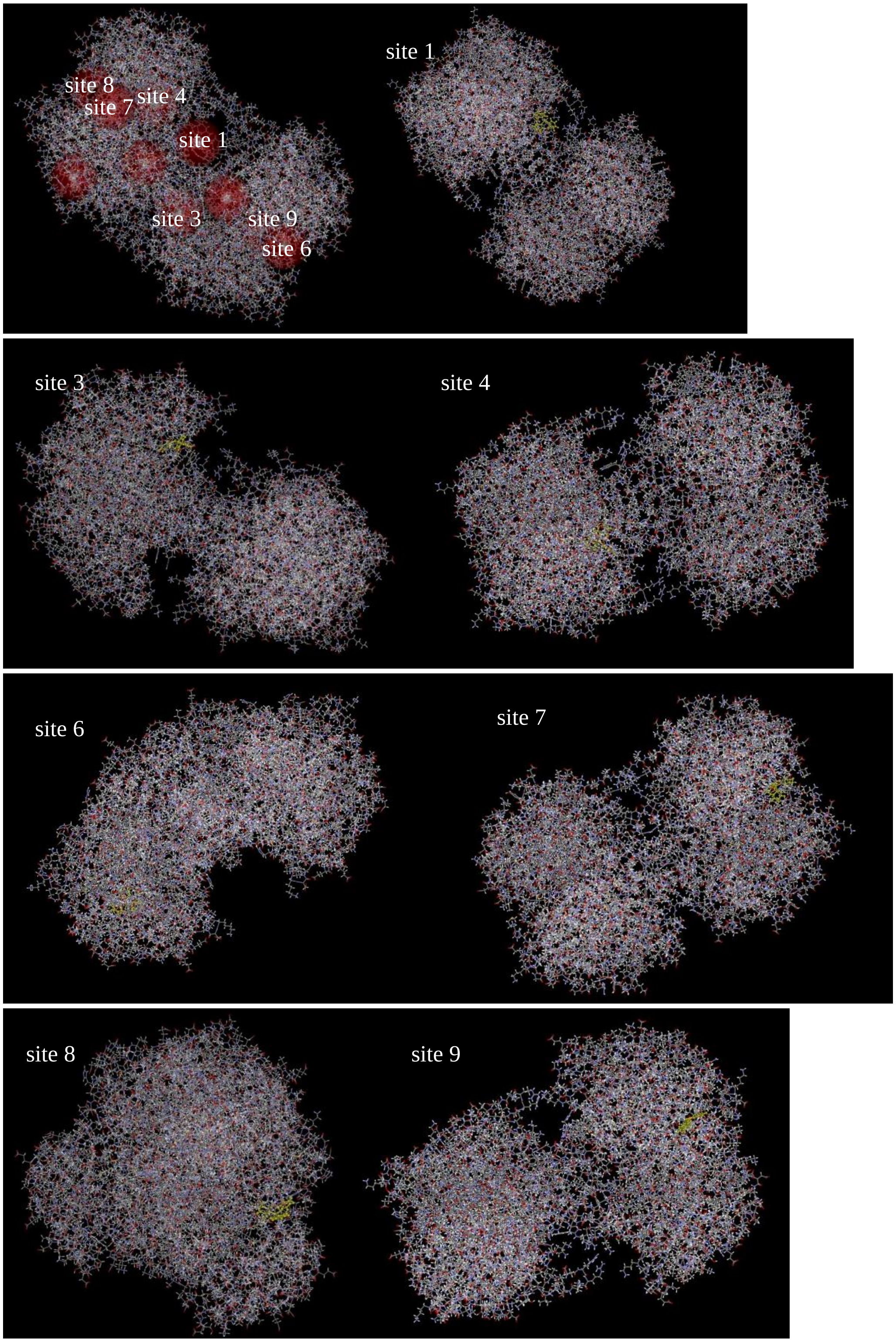

acetyl-CoA acetyltransferase (gi|118418455)-Swiss modle

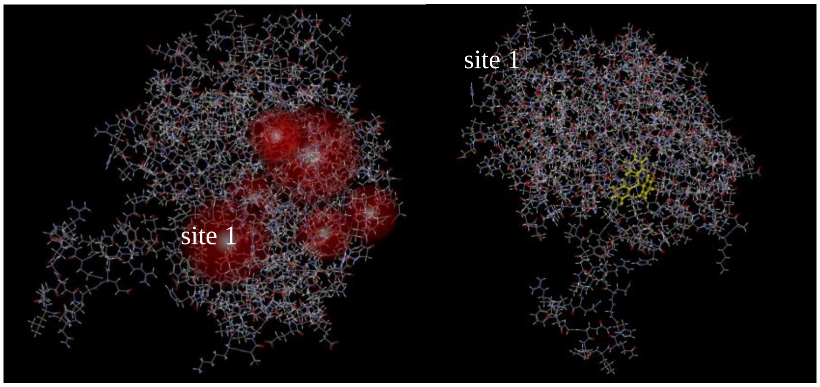

acetyl-CoA acetyltransferase (gi|118418455)-Phyre^{2}

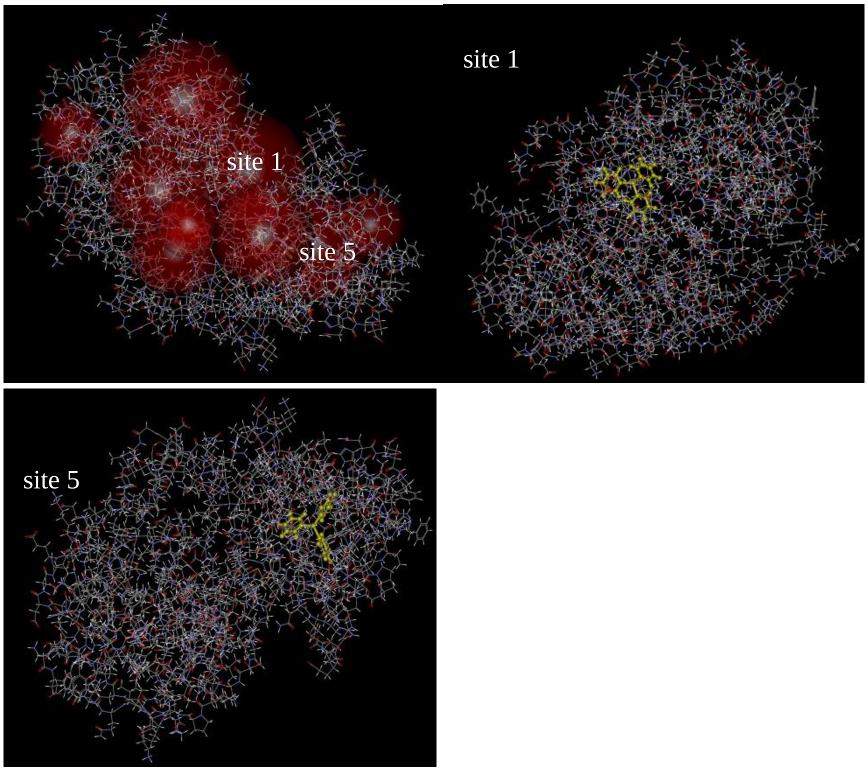

alkyl hydroperoxide reductase C22 (gi|326938119)-Swiss modle

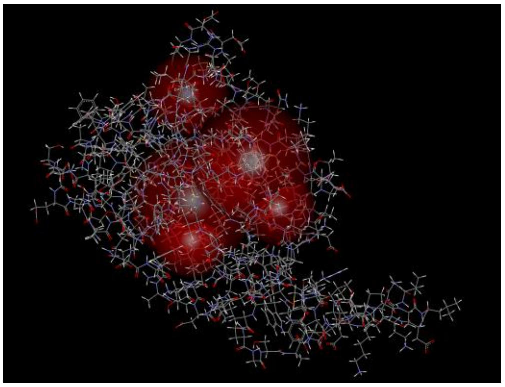

alkyl hydroperoxide reductase C22 (gi|326938119)-Phyre^2

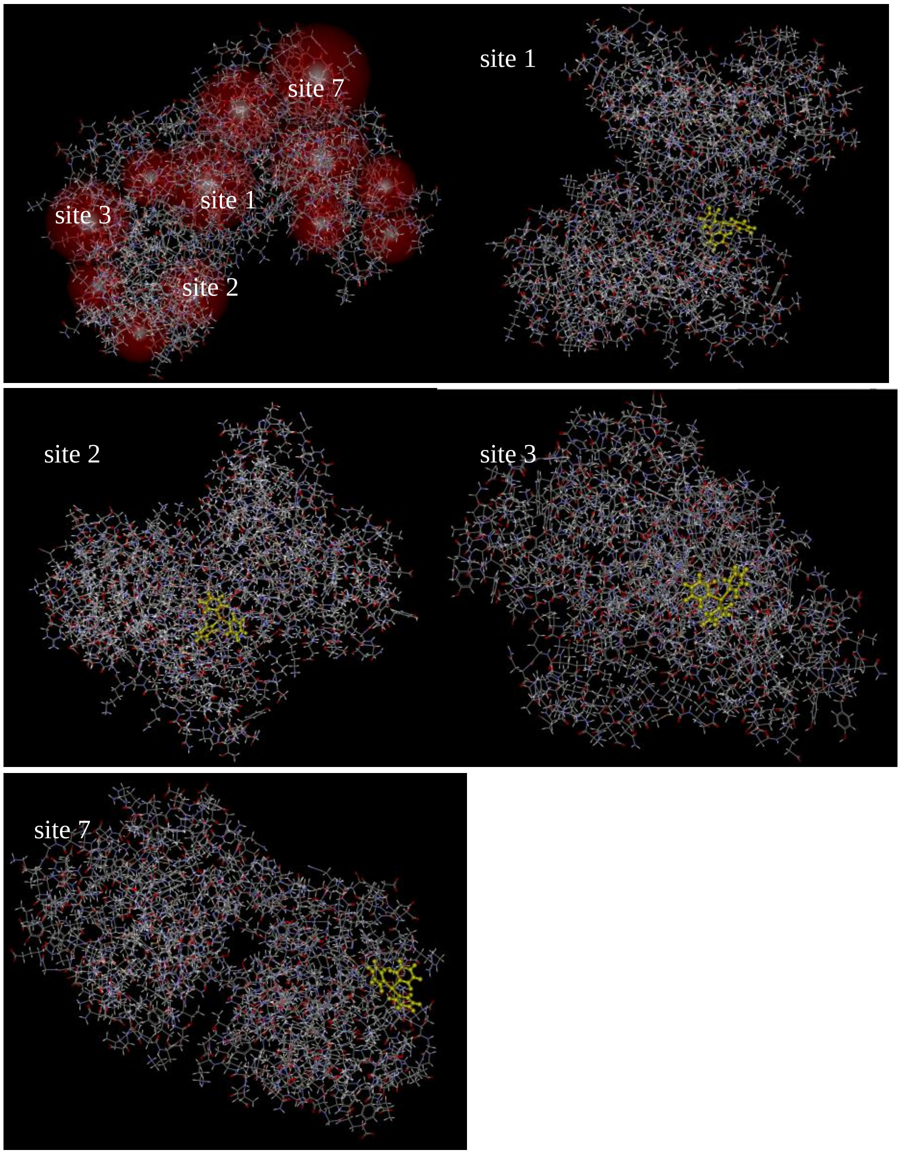

SOD (gi|449026274)-Swiss modle

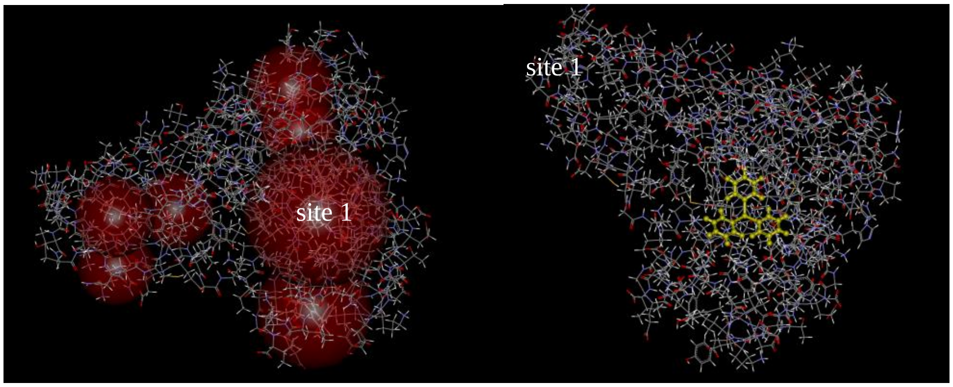

SOD (gi|449026274)-Phyre[2]

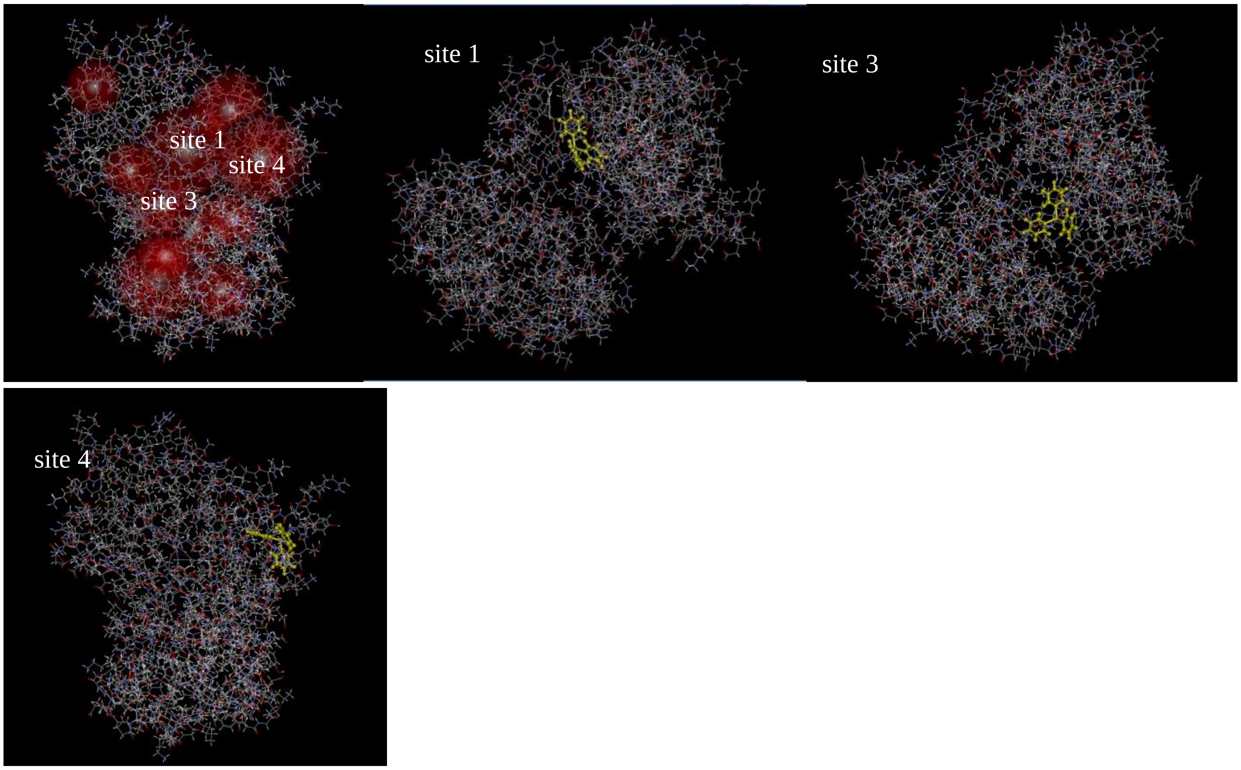

leucine dehydrogenase (gi|449024882)-Swiss modle

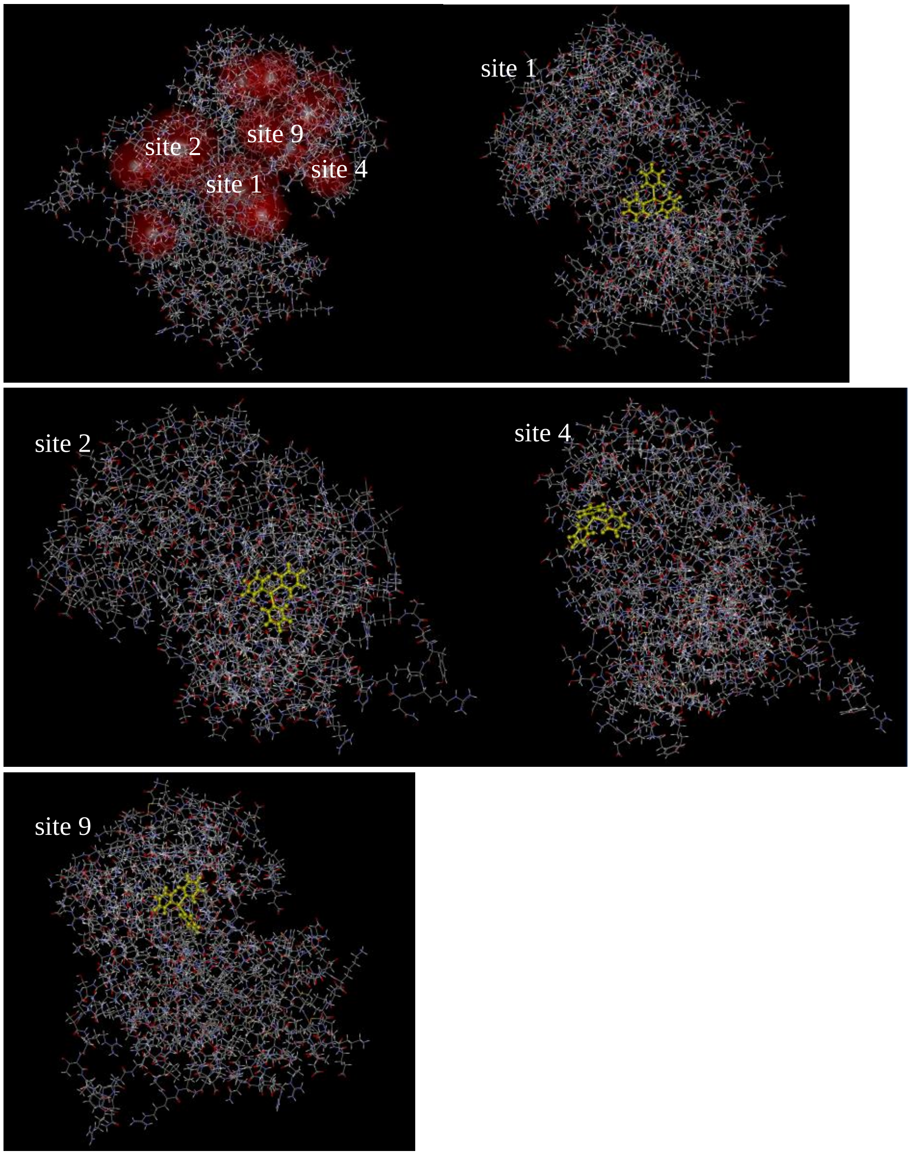

leucine dehydrogenase (gi|449024882)-Phyre^2

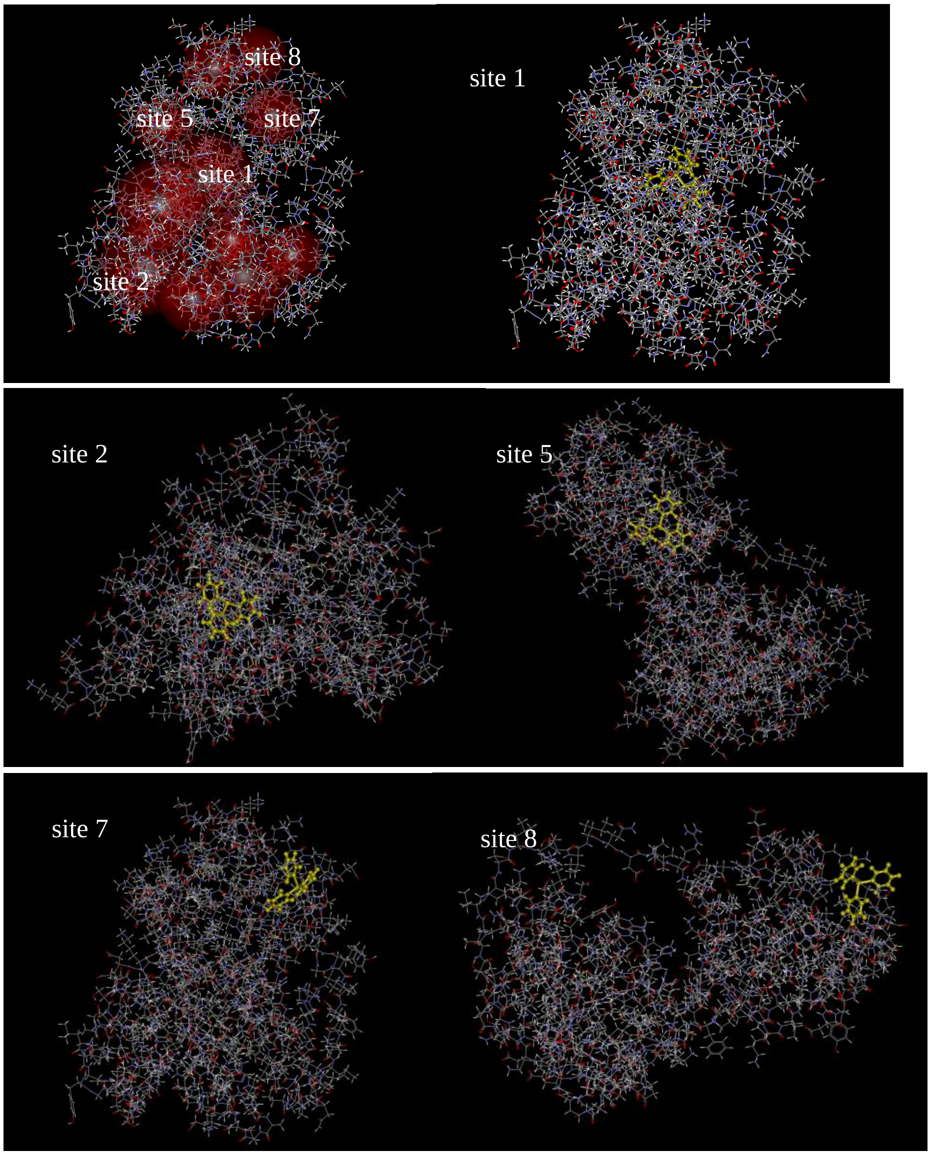

electron transfer flavoprotein subunit alpha (gi|401792614)-Swiss modle

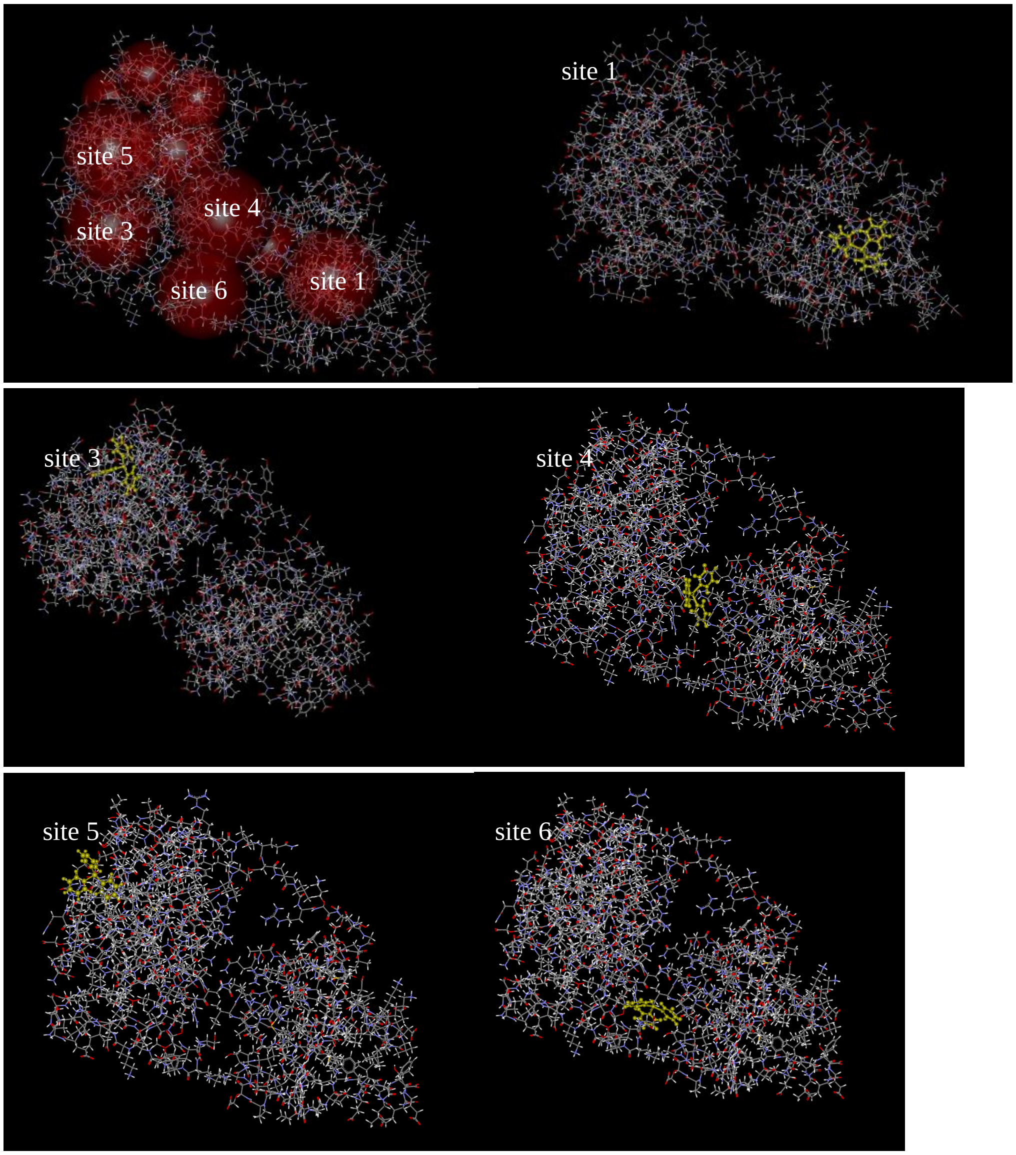

electron transfer flavoprotein subunit alpha (gi|401792614)-Phyre^2

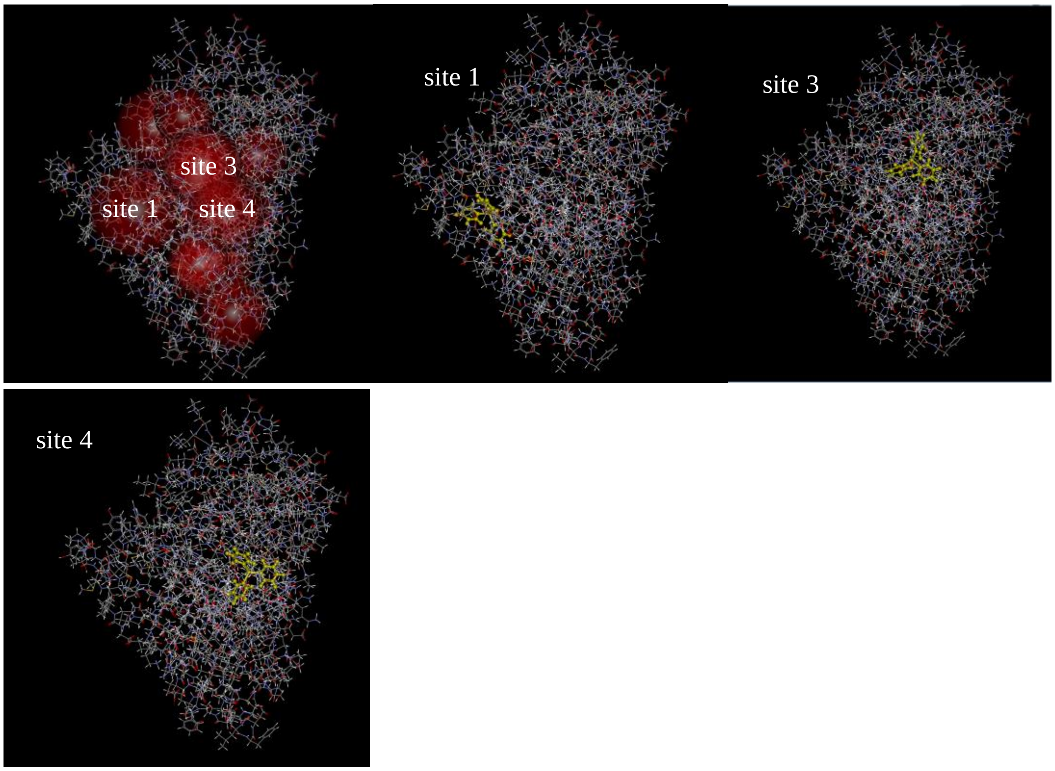

branched-chain alpha-keto acid dehydrogenase E1 component (gi|118418579)-Swiss modle

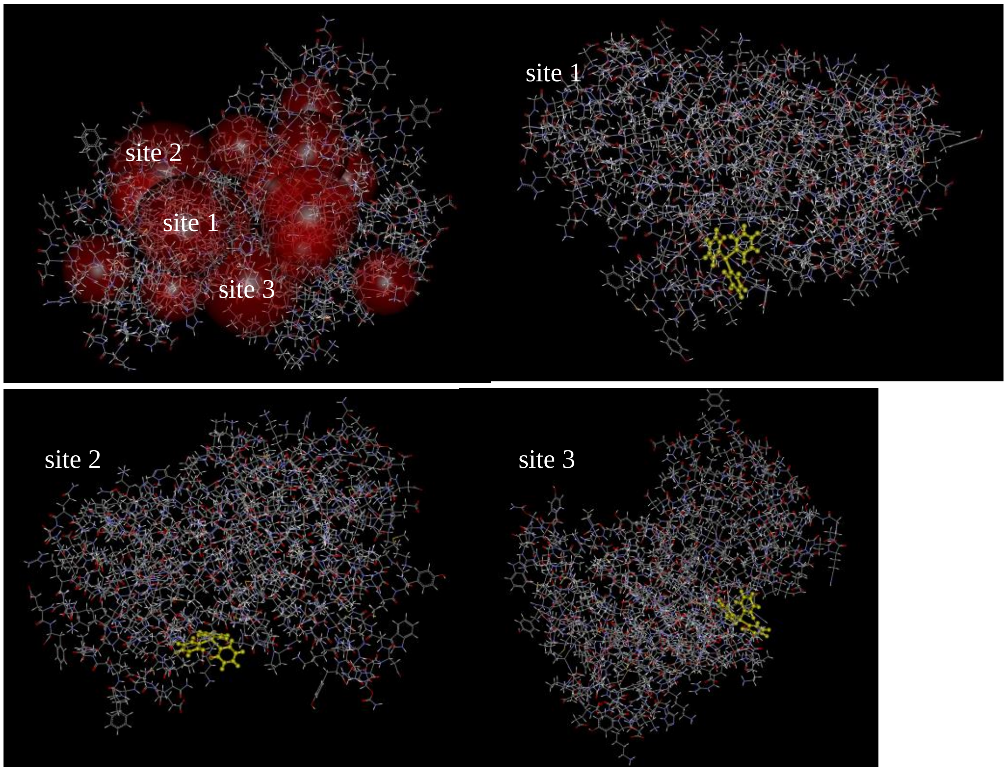

branched-chain alpha-keto acid dehydrogenase E1 component (gi|118418579)-Phyre[2]

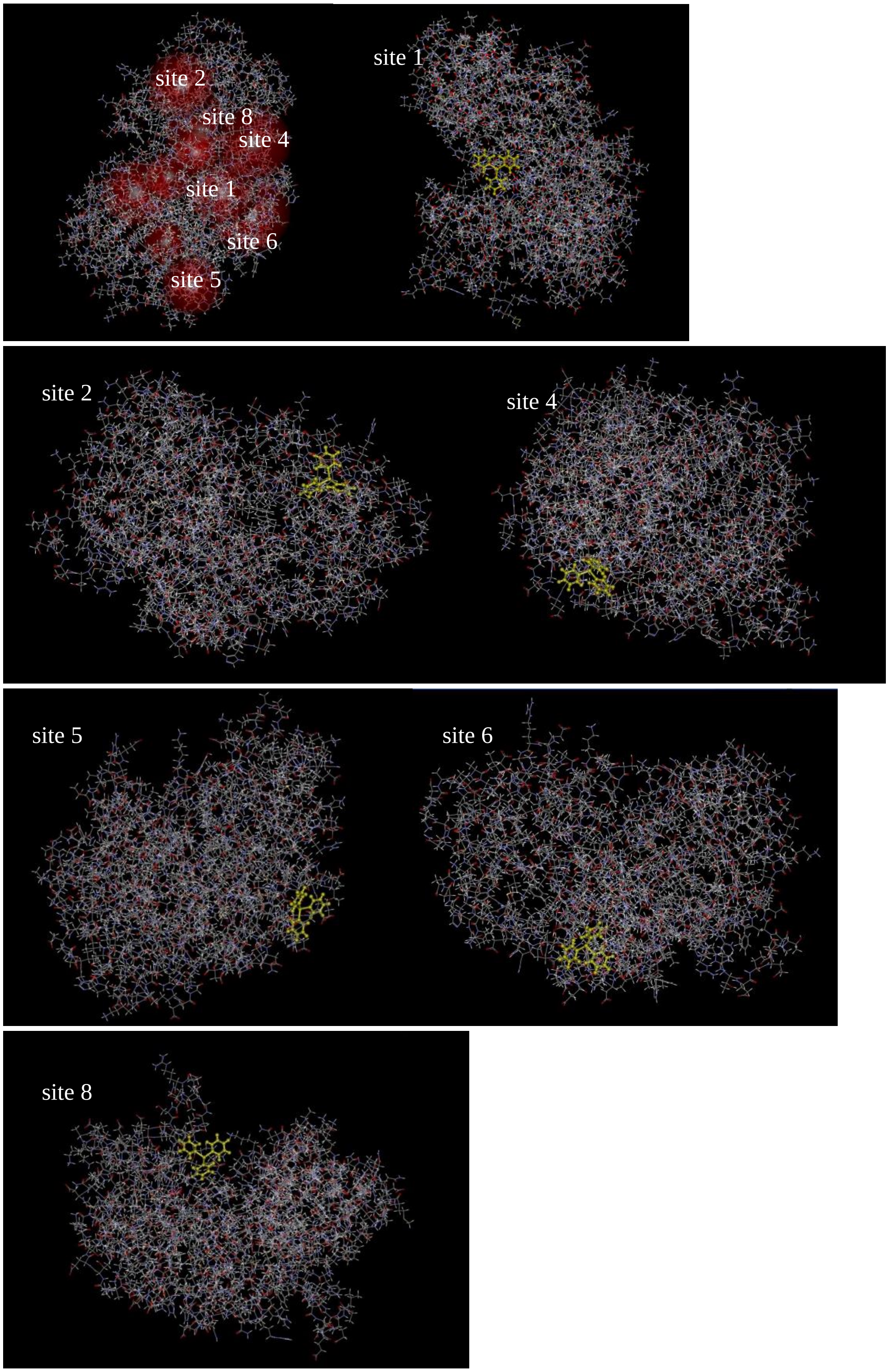

phosphoenolpyruvate carboxykinase (gi|296326283)-Swiss modle

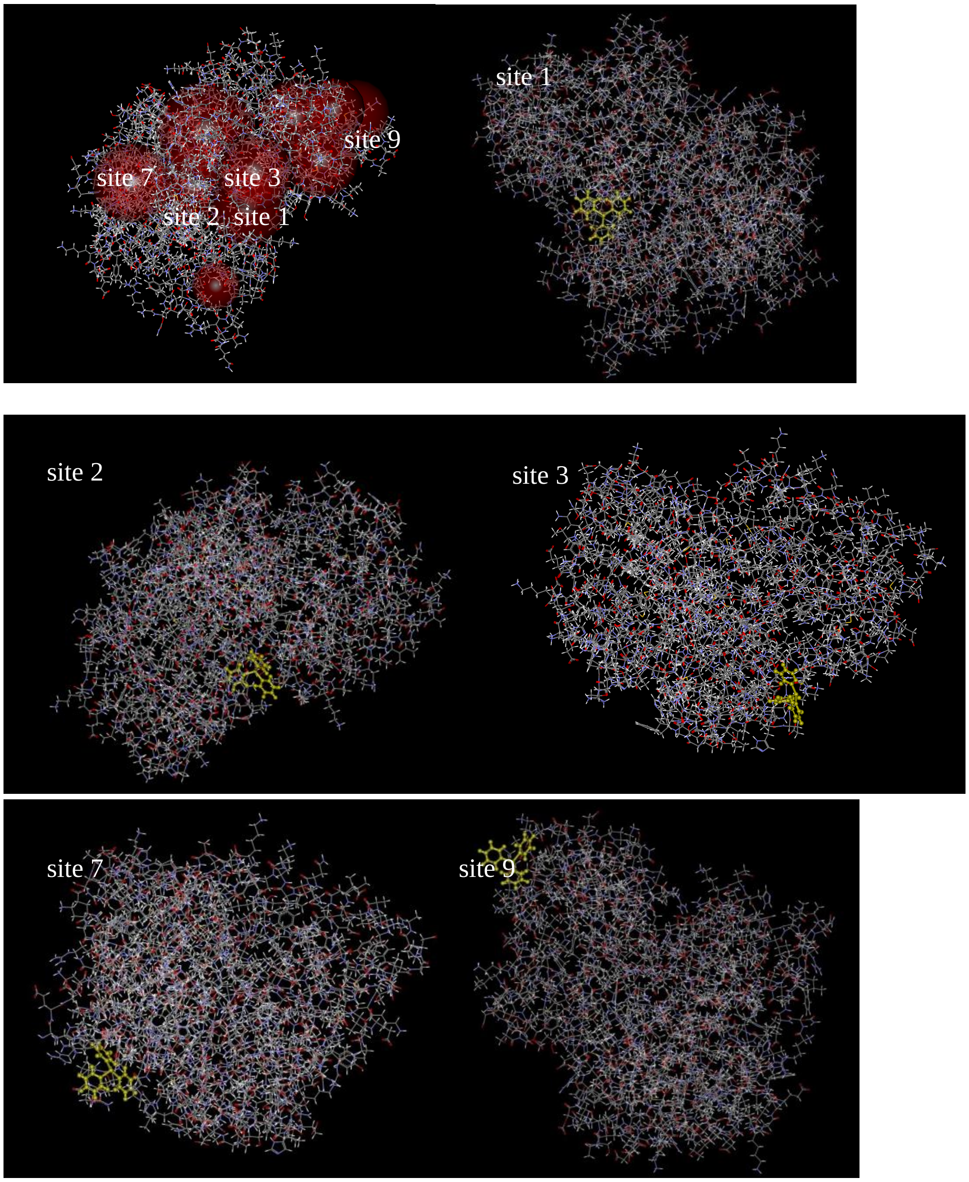

phosphoenolpyruvate carboxykinase (gi|296326283)-Phyre^2

Supplementary Figure S4. TPT binding sites of proteins.

	Ineffective proteins			Target proteins		
	**Percentage**	**Weight**	**Sum**	**Percentage**	**Weight**	**Sum**
ALA	7.6	61.6	469.8	10.4	61.6	637.9
CYS	0.7	16.9	12.3	7.8	16.9	131.0
ASP	4.8	13.8	65.5	0.0	13.8	0.0
GLU	7.1	7.4	52.7	1.0	7.4	7.7
PHE	4.6	4.8	22.0	10.1	4.8	48.4
GLY	7.3	5.4	39.6	6.1	5.4	33.4
HIS	2.0	7.2	14.1	9.4	7.2	68.0
ILE	8.2	5.5	44.9	0.0	5.5	0.0
LYS	7.2	30.7	221.5	5.7	30.7	174.6
LEU	9.3	25.1	232.3	3.9	25.1	98.1
MET	2.9	103.9	302.6	4.1	103.9	431.0
ASN	4.2	8.1	34.1	4.6	8.1	37.3
PRO	3.6	3.4	12.2	7.5	3.4	25.3
GLN	3.3	5.5	18.0	3.1	5.5	17.0
ARG	3.8	6.3	24.1	4.8	6.3	30.1
SER	5.8	14.0	81.5	9.5	14.0	132.8
THR	5.4	2.8	15.3	3.5	2.8	9.8
VAL	7.9	16.9	134.0	6.3	16.9	106.9
TRP	0.8	7.7	6.5	0.0	7.7	0.0
TYR	3.5	1.2	4.1	2.2	1.2	2.6
Sum			1807.1			1992.1

Supplementary Table S1. Relation between TPT recognition and AA compositions of target proteins and ineffective proteins.

Spot No.	Accession No.	Protein Name	Fold	Score (%)
1	gi|296325172	aconitate hydratase [*Bacillus thuringiensis* BMB171]	-4.0	100
2	gi|296325172	aconitate hydratase [*Bacillus thuringiensis* BMB171]	-3.1	100
3	gi|326941332	aconitate hydratase [*Bacillus thuringiensis serovar chinensis* CT-43]	-3.9	100
4	gi|449020530	elongation factor G 2 [*Bacillus thuringiensis serovar kurstaki str*. HD73]	-3.4	100
5	gi|449020530	elongation factor G 2 [*Bacillus thuringiensis serovar kurstaki str*. HD73]	-6.8	100
6	gi|449020530	elongation factor G 2 [*Bacillus thuringiensis serovar kurstaki str*. HD73]	-4.4	100
7	gi|49329881	transketolase [*Bacillus thuringiensis serovar konkukian str*. 97-27]	-2.8	100
8	gi|409172717	immune inhibitor A [*Bacillus thuringiensis* Bt407]	-4.2	100
9	gi|449024556	putative phosphatase [*Bacillus thuringiensis serovar kurstaki str*. HD73]	-4.5	100
10	gi|326937953	D-fructose-6-phosphate amidotransferase [*Bacillus thuringiensis serovar chinensis* CT-43]	-6.5	100
11	gi|326937953	D-fructose-6-phosphate amidotransferase [*Bacillus thuringiensis serovar*	-6.5	100

		chinensis CT-43]		
12	gi|324328938	acyl-CoA dehydrogenase [*Bacillus thuringiensis serovar finitimus* YBT-020]	-2.8	100
13	gi|401876898	phosphomethylpyrimidine synthase ThiC [*Bacillus thuringiensis* HD-789]	-2.0	100
14	a:gi|118417172<br>b:gi|401788842	propionyl-CoA carboxylase beta chain [*Bacillus thuringiensis* HD-771]	2.1	100
15	gi|401872679	hypothetical protein BTF1_03115 [*Bacillus thuringiensis* HD-789]	4.2	100
16	gi|326938118	alkyl hydroperoxide reductase subunit F [*Bacillus thuringiensis serovar chinensis* CT-43]	2.1	100
17	gi|326938118	alkyl hydroperoxide reductase subunit F [*Bacillus thuringiensis serovar chinensis* CT-43]	-3.4	100
18	gi|118415317	alkyl hydroperoxide reductase, subunit F [*Bacillus thuringiensis str. Al Hakam*]	-3.8	100
19	gi|118418112	urocanate hydratase [*Bacillus thuringiensis str. Al Hakam*]	-3.1	100
20	gi|296326283	phosphoenolpyruvate carboxykinase [*Bacillus thuringiensis* BMB171]	-3.8	100
21	gi|326938083	1-pyrroline-5-carboxylate dehydrogenase [*Bacillus thuringiensis serovar chinensis* CT-43]	-2.4	100
22	gi|449022992	methylmalonate semialdehyde dehydrogenase [*Bacillus thuringiensis serovar kurstaki*	-6.0	100

		str. HD73]		
23	gi|449022992	methylmalonate semialdehyde dehydrogenase [*Bacillus thuringiensis serovar kurstaki str*. HD73]	-6.0	100
24	gi|49330832	protein-synthesizing GTPase (translation elongation factor Tu (EF-TU)) [*Bacillus thuringiensis sero var konkukian str*. 97-27]	-6.7	100
25	gi|49330832	protein-synthesizing GTPase (translation elongation factor Tu (EF-TU)) [*Bacillus thuringiensis sero var konkukian str*. 97-27]	-6.7	100
26	gi|409173970	biotin carboxylase 2 [*Bacillus thuringiensis* Bt407]	-3.0	100
27	gi|49333293	DNA-directed RNA polymerase [*Bacillus thuringiensis serovar konkukian str*. 97-27]	-3.1	100
28	gi|324328485	isocitrate dehydrogenase [*Bacillus thuringiensis serovar finitimus* YBT-020]	-4.9	100
29	gi|449024882	leucine dehydrogenase [*Bacillus thuringiensis serovar kurstaki str*. HD73]	-4.6	100
30	gi|118418455	acetyl-CoA acetyltransferase [*Bacillus thuringiensis str. Al Hakam*]	-4.6	100
31	gi|118418759	cystathionine gamma-lyase [*Bacillus thuringiensis str. Al Hakam*]	-4.0	100
32	gi|449022994	enoyl-CoA hydratase [*Bacillus thuringiensis serovar kurstaki str*. HD73]	3.2	100

33	gi|449022994	enoyl-CoA hydratase [*Bacillus thuringiensis serovar kurstaki str*. HD73]	-2.3	100
34	gi|449024916	X-Pro dipeptidase [*Bacillus thuringiensis serovar kurstaki str*. HD73]	-4.0	100
35	gi|401787490	succinyl-CoA synthetase subunit beta [*Bacillus thuringiensis* HD-771]	-5.7	100
36	gi|118415960	aminotransferase [*Bacillus thuringiensis str. Al Hakam*]	-5.7	100
37	gi|296325382	succinyl-CoA synthetase subunit alpha [*Bacillus thuringiensis* BMB171]	-6.3	100
38	gi|118418833	rod shape-determining protein MreB [*Bacillus thuringiensis str. Al Hakam*]	5.0	100
39	gi|118419594	fructose-bisphosphate aldolase [*Bacillus thuringiensis str. Al Hakam*]	-8.4	100
40	gi|118419594	fructose-bisphosphate aldolase [*Bacillus thuringiensis str. Al Hakam*]	-3.1	100
41	gi|401788500	manganese-dependent inorganic pyrophosphatase [*Bacillus thuringiensis* HD-771]	3.7	100
42	gi|118419214	L-lactate dehydrogenase [*Bacillus thuringiensis str. Al Hakam*]	2.7	100
43	gi|296326472	ABC transporter ATP-binding protein [*Bacillus thuringiensis* BMB171]	3.0	100
44	gi|296326472	ABC transporter ATP-binding protein [*Bacillus thuringiensis*	-3.2	100

		BMB171]		
45	gi|118418579	branched-chain alpha-keto acid dehydrogenase E1 component [*Bacillus thuringiensis str. Al Hakam*]	2.5	100
46	gi|296323943	methylisocitrate lyase [*Bacillus thuringiensis* BMB171]	-7.8	100
47	gi|118418579	branched-chain alpha-keto acid dehydrogenase E1 component [*Bacillus thuringiensis str. Al Hakam*]	-8.9	100
48	gi|401788493	dihydrodipicolinate synthase [*Bacillus thuringiensis* HD-771]	3.0	100
49	gi|5542485	chain A, crystal structure of hew lysozyme under pressure of krypton (55 Bar)	-2.3	100
50	gi|449020437	pyridoxal biosynthesis lyase PdxS [*Bacillus thuringiensis serovar kurstaki str*. HD73]	-7.2	100
51	gi|324324774	thiazole synthase [*Bacillus thuringiensis serovar finitimus* YBT-020]	-2.9	100
52	gi|326942228	cell division inhibitor MinD [*Bacillus thuringiensis serovar chinensis* CT-43]	2.7	100
53	gi|326937948	arginase [*Bacillus thuringiensis serovar chinensis* CT-43]	2.1	100
54	gi|326941596	transcriptional repressor CodY [*Bacillus thuringiensis serovar chinensis* CT-43]	-2.1	100
55	gi|449020555	adenylate kinase [*Bacillus thuringiensis serovar kurstaki*	4.2	100

		str. HD73]		
56	gi|296322525	transcriptional activator tenA [*Bacillus thuringiensis* BMB171]	2.6	99
57	gi|381147975	triosephosphate isomerase, partial [*Bacillus thuringiensis serovar kurstaki*]	-9.7	100
58	gi|381147975	triosephosphate isomerase, partial [*Bacillus thuringiensis serovar kurstaki*]	-4.6	100
59	gi|326938119	Alkyl hydroperoxide reductase C22 [*Bacillus thuringiensis serovar chinensis* CT-43]	-10.4	100
60	gi|326938119	Alkyl hydroperoxide reductase C22 [*Bacillus thuringiensis serovar chinensis* CT-43]	-8.2	100
61	gi|326938119	Alkyl hydroperoxide reductase C22 [*Bacillus thuringiensis serovar chinensis* CT-43]	2.1	100

Supplementary Table S2. Summary of the identified proteins with different expressions after interaction with TPT in a polyacrylamide gel during the silver staining process.

Spot No.	Accession No.	Protein Name	Fold	Score (%)
1	gi|296325172	aconitate hydratase [*Bacillus thuringiensis* BMB171]	-3.4	100
2	gi|449024305	aconitate hydratase [*Bacillus thuringiensis serovar kurstaki str*. HD73]	-4.1	100
3	gi|296325172	aconitate hydratase [*Bacillus thuringiensis* BMB171]	-3.6	100
4	gi|401876112	alanyl-tRNA ligase [*Bacillus thuringiensis* HD-789]	4.5	100
5	gi|449020530	elongation factor G 2 [*Bacillus thuringiensis serovar kurstaki str*. HD73]	-4.3	100
6	gi|118418436	methionine synthase (B12-independent) [*Bacillus thuringiensis str. Al Hakam*]	-3.0	100
7	gi|118418436	methionine synthase (B12-independent) [*Bacillus thuringiensis str. Al Hakam*]	-4.4	100
8	gi|449022990	acyl-CoA dehydrogenase [*Bacillus thuringiensis serovar kurstaki str*. HD73]	-7.8	100
9	gi|449022990	acyl-CoA dehydrogenase [*Bacillus thuringiensis serovar kurstaki str*. HD73]	-3.1	100
10	gi|118418220	pyruvate ferredoxin oxidoreductase, alpha subunit [*Bacillus thuringiensis str. Al Hakam*]	-3.1	100
11	gi|296326283	phosphoenolpyruvate carboxykinase [*Bacillus thuringiensis* BMB171]	-3.2	100
12	gi|409173970	biotin carboxylase 2 [*Bacillus*	-4.0	100

		thuringiensis Bt407]		
13	gi|409175007	putative aldehyde dehydrogenase DhaS [*Bacillus thuringiensis* Bt407]	-3.1	100
14	gi|449024882	leucine dehydrogenase [*Bacillus thuringiensis serovar kurstaki str*. HD73]	-3.2	100
15	gi|296325759	glycine cleavage system aminomethyltransferase T [*Bacillus thuringiensis* BMB171]	-3.6	100
16	a:gi|449022922<br>b:gi|296323882	a:putative alcohol dehydrogenase, zinc-containing [*Bacillus thuringiensis serovar kurstaki str*. HD73]<br>b:alcohol dehydrogenase [*Bacillus thuringiensis* BMB171]	5.0	100
17	gi|118418282	succinyl-CoA synthetase (ADP-forming) alpha subunit [*Bacillus thuringiensis str. Al Hakam*]	-2.5	100
18	gi|49329954	translation elongation factor Ts [*Bacillus thuringiensis serovar konkukian str*. 97-27]	-2.7	100
19	gi|296323941	citrate synthase 3 [*Bacillus thuringiensis* BMB171]	-2.3	100
20	gi|118418579	branched-chain alpha-keto acid dehydrogenase E1 component [*Bacillus thuringiensis str. Al Hakam*]	-3.0	100
21	gi|401792614	electron transfer flavoprotein subunit alpha [*Bacillus thuringiensis* HD-771]	-3.0	100
22	gi|401792614	electron transfer flavoprotein subunit alpha [*Bacillus thuringiensis* HD-771]	-3.0	100
23	gi|326943141	3-hydroxybutyryl-CoA	2.3	100

		dehydrogenase [*Bacillus thuringiensis serovar chinensis* CT-43]		
24	gi|401790890	bifunctional hydroxy-methylpyrimidine kinase/ hydroxy-phosphomethylpyrimidine kinase [*Bacillus thuringiensis* HD-771]	-2.0	99
25	gi|449025585	naphthoate synthase [*Bacillus thuringiensis serovar kurstaki str*. HD73]	2.6	100
26	gi|449026274	superoxide dismutase [*Bacillus thuringiensis serovar kurstaki str*. HD73]	-2.9	100
27	gi|409172358	putative enoyl-CoA hydratase/isomerase YhaR [*Bacillus thuringiensis* Bt407]	2.1	100
28	gi|407385344	ribosome-associated factor Y [*Bacillus thuringiensis* MC28]	-3.1	100
29	gi|407385344	ribosome-associated factor Y [*Bacillus thuringiensis* MC28]	-5.8	100
30	gi|326938119	alkyl hydroperoxide reductase C22 [*Bacillus thuringiensis serovar chinensis* CT-43]	2.1	100
31	gi|326938119	alkyl hydroperoxide reductase C22 [*Bacillus thuringiensis serovar chinensis* CT-43]	-2.0	100
32	gi|326938119	alkyl hydroperoxide reductase C22 [*Bacillus thuringiensis serovar chinensis* CT-43]	-3.0	100
33	gi|326938119	alkyl hydroperoxide reductase C22 [*Bacillus thuringiensis serovar chinensis* CT-43]	5.0	100
34	gi|326938119	alkyl hydroperoxide reductase C22 [*Bacillus thuringiensis*	4.0	100

		serovar chinensis CT-43]		
35	gi|49332686	ATP-dependent Clp protease, proteolytic subunit (endopeptidase Clp) [[*Bacillus thuringiensis] serovar konkukian str.* 97-27]	7.7	100
36	gi|118418342	cell-division initiation protein (divIVA) [*Bacillus thuringiensis str. Al Hakam*]	6.9	100
37	gi|296321985	50S ribosomal protein L10 [*Bacillus thuringiensis* BMB171]	2.5	100

Supplementary Table S3. Summary of the identified proteins with different expressions after interaction with TPT in a low melting point agarose gel and a polyacrylamide gel during the vertical electrophoresis process.

Spot No.	Accession No.	Protein name	Fold	Score (%)
1	gi|401791452	elongation factor Tu [*Bacillus thuringiensis* HD-771]	-4.0	100
2	gi|326941397	transketolase [*Bacillus thuringiensis serovar chinensis* CT-43]	-2.5	100
3	gi|407383748	transketolase [*Bacillus thuringiensis* MC28]	-2.3	100
4	gi|326937953	D-fructose-6-phosphate amidotransferase [*Bacillus thuringiensis serovar chinensis* CT-43]	2.5	100
5	gi|49332762	adenylosuccinate synthase (IMP--aspartate ligase) [*Bacillus thuringiensis serovar konkukian str.* 97-27]	2.3	100
6	gi|118417172	propionyl-CoA carboxylase [*Bacillus thuringiensis str. Al Hakam*]	-2.9	100
7	gi|118417172	propionyl-CoA carboxylase [*Bacillus thuringiensis str. Al Hakam*]	3.2	100
8	gi|449020435	inosine 5'-monophosphate dehydrogenase [*Bacillus thuringiensis serovar kurstaki str*. HD73]	-5.2	100
9	gi|326937805	inositol-5-monophosphate dehydrogenase [*Bacillus thuringiensis serovar chinensis* CT-43]	4.2	100
10	gi|449024218	glyoxalase [*Bacillus thuringiensis serovar kurstaki str*. HD73]	-2.1	100
11	gi|118419594	fructose-bisphosphate aldolase [*Bacillus thuringiensis str. Al*	-2.9	100

		Hakam]		
12	gi|449025847	pyridine nucleotide-disulfide oxidoreductase [*Bacillus thuringiensis serovar kurstaki str*. HD73]	-2.9	100
13	gi|401792614	electron transfer flavoprotein subunit alpha [*Bacillus thuringiensis* HD-771]	-2.9	100
14	gi|401792614	electron transfer flavoprotein subunit alpha [*Bacillus thuringiensis* HD-771]	-2.9	100
15	gi|401792614	electron transfer flavoprotein subunit alpha [*Bacillus thuringiensis* HD-771]	-2.9	100
16	gi|407385686	ribose-phosphate pyrophosphokinase 1 [*Bacillus thuringiensis* MC28]	2.4	100
17	gi|449026274	superoxide dismutase [*Bacillus thuringiensis serovar kurstaki str*. HD73]	4.0	100
18	gi|449026274	superoxide dismutase [*Bacillus thuringiensis serovar kurstaki str*. HD73]	-5.0	100
19	gi|296323718	CBS domain-containing protein [*Bacillus thuringiensis* BMB171]	3.9	100
20	gi|49332686	ATP-dependent Clp protease, proteolytic subunit (endopeptidase Clp) [*Bacillus thuringiensis serovar konkukian str*. 97-27]	2.6	100
21	gi|407385344	ribosome-associated factor Y [*Bacillus thuringiensis* MC28]	2.9	100
22	gi|407385344	ribosome-associated factor Y [*Bacillus thuringiensis* MC28]	4.4	100

23	gi|326942307	enoyl-CoA hydratase [*Bacillus thuringiensis serovar chinensis* CT-43]	4.9	100

Supplementary Table S4. Summary of the identified proteins with different expressions after interaction with TPT in solution.

No.	Unused score	Accession No.	Protein name	**117:114** (treatment sample: control sample)
1	61.3	gi|595878184	aconitate hydratase	2.6
2	58.7	gi|944305122	formate acetyltransferase	4.4
3	55.2	gi|958622442	1-pyrroline-5-carboxylate dehydrogenase	2.9
4	48.1	gi|942010301	pyruvate carboxylase	2.2
5	42.5	gi|940630194	molecular chaperone GroEL	4.9
6	42.5	gi|595876612	betaine-aldehyde dehydrogenase	8.6
7	41.0	gi|958622268	elongation factor G	1.5
8	39.6	gi|595878916	flagellin	0.7
9	39.0	gi|595879831	ATP F0F1 synthase subunit beta	1.1
10	38.6	gi|940631722	succinyl-CoA synthetase subunit beta	5.3
11	38.3	gi|944303324	ethanol-active dehydrogenase/acetaldehyde-active reductase	3.5
12	36.8	gi|958626454	isocitrate dehydrogenase	5.2
13	35.1	gi|595878156	peptide ABC transporter substrate-binding protein	1.1
14	33.3	gi|595880386	alanine dehydrogenase	2.7
15	33.2	gi|958624436	acyl-CoA dehydrogenase	3.3
16	33.1	gi|958622269	elongation factor Tu	
17	32.4	gi|940632258	enolase	1.9
18	32.2	gi|958622242	ATP-dependent Clp protease ATP-binding subunit ClpC	2.9

19	31.9	gi|942011367	acyl-CoA dehydrogenase	4.4
20	30.1	gi|940629231	inosine-5'-monophosphate dehydrogenase	1.1
21	28.4	gi|944305204	arginine deiminase	0.4
22	27.4	gi|944303254	methylmalonate-semialdehyde dehydrogenase	3.1
23	26.2	gi|958624441	carboxylase	5.1
24	25.8	gi|958625851	dihydrolipoamide dehydrogenase	0.6
25	25.5	gi|944304211	3-oxoacyl-ACP synthase	5.2
26	25.3	gi|944303258	2-methylcitrate dehydratase	1.2
27	24.8	gi|942011624	ATP synthase subunit alpha	2.4
28	24.6	gi|958626049	leucine dehydrogenase	0.7
29	24.6	gi|595876581	peptide ABC transporter substrate-binding protein	0.1
30	24.1	gi|942010192	succinyl-CoA synthetase subunit alpha	3.8
31	22.9	gi|944305233	alkyl hydroperoxide reductase	3.7
32	21.6	gi|944304210	poly(R)-hydroxyalkanoic acid synthase	10.9
33	21.4	gi|662062902	phosphoenolpyruvate carboxykinase (ATP)	1.2
34	21.4	gi|958626491	universal stress protein UspA	8.1
35	21.3	gi|942010913	electron transfer flavoprotein subunit alpha	2.5
36	21.2	gi|944303574	hypothetical protein AQ980_19875	8.4
37	21.2	gi|595875828	cysteine synthase	1.0
38	21.1	gi|595880415	Clp protease ATPase	0.9
39	21.1	gi|958626192	molecular chaperone DnaK	1.3
40	20.9	gi|942010579	glycine cleavage system protein T	0.7
41	20.7	gi|940629570	glycine dehydrogenase	1.2

42	20.4	gi|595879842	serine hydroxymethyltransferase	1.2
43	20.2	gi|958622625	flotillin	0.8
44	19.8	gi|595876031	pyridine nucleotide-disulfide oxidoreductase	3.1
45	19.6	gi|940629634	electron transfer flavoprotein subunit beta	3.1
46	19.2	gi|958625708	elongation factor Ts	0.9
47	19.1	gi|958623199	ornithine--oxo-acid aminotransferase	1.1
48	19.1	gi|958622263	DNA-directed RNA polymerase subunit beta	1.1
49	18.5	gi|940629633	enoyl-CoA hydratase	3.3
50	18.4	gi|944304214	DNA recombinase	24.4
51	18.4	gi|940630882	polyhydroxyalkanoate biosynthesis repressor PhaR	11.1
52	18.0	gi|958625852	branched-chain alpha-keto acid dehydrogenase subunit E2	0.6
53	17.7	gi|595877435	DNA-directed RNA polymerase subunit beta'	1.1
54	17.6	gi|958625853	2-oxoisovalerate dehydrogenase	0.9
55	17.5	gi|595878530	succinate dehydrogenase	2.3
56	17.2	gi|942011470	glyceraldehyde-3-phosphate dehydrogenase	4.1
57	16.9	gi|595877416	50S ribosomal protein L5	2.9
58	16.5	gi|595879634	glycine dehydrogenase	1.2
59	16.4	gi|958622619	protein prkA	0.0
60	16.4	gi|942012037	alkyl hydroperoxide reductase subunit F	1.0
61	16.4	gi|595879901	phosphoglycerate kinase	0.5
62	16.0	gi|940631607	pyruvate dehydrogenase (acetyl-transferring) E1 component subunit	1.0

			alpha	
63	15.9	gi|940630150	formate--tetrahydrofolate ligase	4.7
64	15.5	gi|958626045	2-oxoisovalerate dehydrogenase	1.0
65	15.2	gi|958622277	30S ribosomal protein S3	2.3
66	15.1	gi|595879698	branched-chain alpha-keto acid dehydrogenase subunit E2	1.0
67	14.5	gi|958622267	30S ribosomal protein S7	3.6
68	14.3	gi|940628920	superoxide dismutase	1.3
69	14.2	gi|958622260	50S ribosomal protein L10	1.0
70	14.1	gi|944304016	nucleoside diphosphate kinase	1.0
71	14.0	gi|595880431	translation initiation factor IF-2	0.9
72	14.0	gi|958626958	ATP-dependent Clp protease proteolytic subunit	6.1
73	13.9	gi|958622288	30S ribosomal protein S5	1.9
74	13.9	gi|958626455	citrate synthase	4.3
75	13.8	gi|944303255	3-hydroxyisobutyrate dehydrogenase	1.0
76	13.8	gi|595879861	fructose-bisphosphate aldolase	1.8
77	13.8	gi|944303352	penicillin-binding protein	5.3
78	13.7	gi|944304040	glutamate dehydrogenase	3.9
79	13.7	gi|595880179	30S ribosomal protein S4	4.3
80	13.5	gi|944304246	cell division protein FtsN	3.8
81	13.4	gi|958625709	30S ribosomal protein S2	1.0
82	13.4	gi|958626378	succinate dehydrogenase	1.0
83	12.9	gi|942010971	glyceraldehyde-3-phosphate dehydrogenase	8.2
84	12.8	gi|595877440	50S ribosomal protein L1	1.0
85	12.8	gi|958623267	enoyl-ACP reductase	3.4

86	12.6	gi|958625907	acetyl-CoA acetyltransferase	2.0
87	12.1	gi|595875149	transketolase	1.0
88	12.0	gi|958626453	malate dehydrogenase	0.9
89	11.8	gi|595880451	hypothetical protein BF15_17725	0.9
90	11.5	gi|958622317	glutamine--fructose-6-phosphate aminotransferase	1.0
91	11.5	gi|958622271	50S ribosomal protein L3	2.2
92	11.5	gi|940632040	ornithine carbamoyltransferase	0.7
93	11.3	gi|958622793	thiazole synthase	2.1
94	11.2	gi|662061834	5-methyltetrahydropteroyltriglutamate--homocysteine S-methyltransferase	0.8
95	11.1	gi|940629084	aminopeptidase	1.4
96	11.1	gi|944305012	8-amino-7-oxononanoate synthase	1.0
97	11.0	gi|958625710	transcriptional repressor CodY	1.2
98	10.8	gi|958622275	30S ribosomal protein S19	0.6
99	10.7	gi|958626676	phage shock protein A	1.0
100	10.5	gi|958626885	peptidase M4	1.4
101	10.5	gi|940631502	hypothetical protein AL712_14480	0.1
102	10.2	gi|942013047	30S ribosomal protein S1	0.9
103	10.2	gi|958626940	triose-phosphate isomerase	1.1
104	10.1	gi|944303330	molecular chaperone Hsp20	4.6
105	10.1	gi|958626383	thioredoxin	0.7
106	10.0	gi|944302469	AMP-dependent synthetase	7.1
107	10.0	gi|958626320	50S ribosomal protein L21	2.5
108	9.9	gi|940628090	glutamine synthetase	3.1
109	9.9	gi|958626046	2-oxoisovalerate dehydrogenase	1.0

110	9.9	gi|940628596	nitroreductase	1.0
111	9.8	gi|595877413	50S ribosomal protein L6	1.2
112	9.7	gi|958622276	50S ribosomal protein L22	1.1
113	9.6	gi|958622285	30S ribosomal protein S8	7.7
114	9.5	gi|595878227	urocanate hydratase	3.4
115	9.4	gi|958626434	peptidase M28	2.1
116	9.4	gi|944305166	chemical-damaging agent resistance protein C	2.5
117	9.4	gi|942010838	50S ribosomal protein L27	1.0
118	9.3	gi|958626347	trigger factor	1.0
119	9.2	gi|944304243	peptidase M6	1.6
120	9.0	gi|958624437	biotin carboxylase	2.9
121	9.0	gi|942011264	L-lactate dehydrogenase	1.8
122	9.0	gi|595877409	50S ribosomal protein L15	1.0
123	9.0	gi|958626346	ATP-dependent Clp protease ATP-binding subunit ClpX	6.4
124	8.8	gi|958626047	dihydrolipoamide dehydrogenase	0.5
125	8.8	gi|958626505	peroxidase	1.1
126	8.7	gi|958625673	4-hydroxy-tetrahydrodipicolinate synthase	1.3
127	8.7	gi|958622450	arsenic-transporting ATPase	2.9
128	8.7	gi|958622299	50S ribosomal protein L17	2.0
129	8.6	gi|595874570	pyridoxal biosynthesis protein	0.8
130	8.6	gi|958627099	ATP synthase F0F1 subunit gamma	1.0
131	8.5	gi|595880214	aminopeptidase	0.7
132	8.4	gi|958622384	4-hydroxyphenylpyruvate dioxygenase	1.1
133	8.4	gi|942010182	ribosome recycling factor	0.8

134	8.3	gi|958622258	50S ribosomal protein L11	3.2
135	8.3	gi|958623551	DNA-binding protein	0.6
136	8.2	gi|595876032	hypothetical protein BF15_26760	1.0
137	8.2	gi|958627124	fructose 1,6-bisphosphatase	0.9
138	8.1	gi|944304881	quinol oxidase subunit 2	0.8
139	8.1	gi|944304856	ABC transporter substrate-binding protein	1.0
140	8.0	gi|958622270	30S ribosomal protein S10	1.1
141	7.9	gi|958622261	50S ribosomal protein L7/L12	0.9
142	7.9	gi|940629453	5'-methylthioadenosine nucleosidase	1.0
143	7.8	gi|958625726	30S ribosomal protein S16	2.4
144	7.7	gi|662063895	heme-binding protein	1.6
145	7.7	gi|944304339	peptide ABC transporter substrate-binding protein	1.1
146	7.7	gi|595879199	30S ribosomal protein S6	4.9
147	7.6	gi|958624248	citrate synthase 3	2.2
148	7.5	gi|958623309	dihydrolipoamide succinyltransferase	1.1
149	7.5	gi|940632276	glmZ(sRNA)-inactivating NTPase	2.5
150	7.4	gi|958622787	thiaminase II	1.0
151	7.4	gi|940631525	peptidylprolyl isomerase	0.7
152	7.4	gi|958623994	DNA starvation/stationary phase protection protein	6.1
153	7.3	gi|595880363	pyruvate kinase	1.2
154	7.2	gi|940629158	3-hydroxyacyl-CoA dehydrogenase	0.5
155	7.1	gi|944304969	peptidase M6	2.1
156	7.1	gi|944303490	type VII secretion protein EsxA	0.5
157	7.0	gi|944304358	ATP-dependent chaperone ClpB	1.5

158	7.0	gi|942011154	S-adenosylmethionine synthetase	0.8
159	7.0	gi|942012470	hypothetical protein BTXL6_14180	1.2
160	6.9	gi|595877410	50S ribosomal protein L30	4.8
161	6.8	gi|958626845	L-lactate dehydrogenase	1.3
162	6.8	gi|940630193	hypothetical protein AL712_22885	4.9
163	6.8	gi|958622303	50S ribosomal protein L13	1.7
164	6.5	gi|958622281	50S ribosomal protein L14	1.0
165	6.5	gi|940631789	aspartate-semialdehyde dehydrogenase	1.1
166	6.5	gi|958622211	ribose-phosphate pyrophosphokinase	0.8
167	6.5	gi|958625722	50S ribosomal protein L19	1.3
168	6.5	gi|958623272	hypothetical protein ATN06_06180	0.0
169	6.4	gi|595879920	thioredoxin reductase	1.1
170	6.4	gi|958626995	Fis family transcriptional regulator	2.3
171	6.3	gi|595874268	glycyl-tRNA ligase	2.1
172	6.3	gi|595877402	30S ribosomal protein S11	0.9
173	6.2	gi|944304547	glycerol kinase	0.8
174	6.2	gi|958623276	hypothetical protein ATN06_06200	3.5
175	6.1	gi|958626248	cystathionine gamma-synthase	1.0
176	6.1	gi|958625863	2,3,4,5-tetrahydropyridine-2,6-dicarboxylate N-acetyltransferase	1.2
177	6.1	gi|958623736	hypothetical protein ATN06_08605	1.2
178	6.1	gi|958624274	DNA-binding protein	1.8
179	6.1	gi|944304248	cell division protein FtsN	1.0
180	6.0	gi|958626029	hypothetical protein ATN06_20890	0.8
181	6.0	gi|940629414	methionine--tRNA ligase	0.6
182	6.0	gi|942010402	hypothetical protein BTXL6_02710	0.1

183	6.0	gi|940631203	cytoplasmic protein	0.4
184	5.8	gi|958626830	Fe-S cluster assembly ATPase SufC	1.3
185	5.8	gi|944305043	alanine dehydrogenase	1.4
186	5.8	gi|940632795	3-hydroxyisobutyryl-CoA hydrolase	0.9
187	5.7	gi|944305158	hypothetical protein AQ980_28370	0.7
188	5.7	gi|958625420	cold-shock protein	0.9
189	5.7	gi|958624155	MecA protein	1.0
190	5.6	gi|942011625	ATP synthase F0F1 subunit delta	4.0
191	5.6	gi|595877403	30S ribosomal protein S13	1.0
192	5.5	gi|944304994	glutamine ABC transporter substrate-binding protein	0.1
193	5.5	gi|942011343	Fe-S cluster assembly protein SufD	0.5
194	5.4	gi|958622298	DNA-directed RNA polymerase subunit alpha	0.9
195	5.4	gi|940629606	bifunctional 5,10-methylene-tetrahydrofolate dehydrogenase/5,10-methylene-tetrahydrofolate cyclohydrolase	1.3
196	5.3	gi|944304022	stage IV sporulation protein A	0.5
197	5.3	gi|958626566	thioredoxin	0.9
198	5.2	gi|958626512	spore protein	0.0
199	5.2	gi|958622225	hypoxanthine phosphoribosyltransferase	1.1
200	5.1	gi|958626749	glucose-6-phosphate isomerase	2.0
201	5.1	gi|944304970	fructose-6-phosphate aldolase	0.6
202	4.8	gi|595880491	hypothetical protein BF15_14820	0.9
203	4.8	gi|944305162	hypothetical protein AQ980_28390	0.4
204	4.8	gi|958625651	stage V sporulation protein S	0.5
205	4.8	gi|942010698	phosphotransferase	1.4

206	4.8	gi|958626508	NAD kinase	1.6
207	4.6	gi|940632627	malate:quinone oxidoreductase	9.2
208	4.6	gi|958622272	50S ribosomal protein L4	1.1
209	4.6	gi|958626438	threonine--tRNA ligase	
210	4.5	gi|940630702	histidinol-phosphate aminotransferase	1.1
211	4.4	gi|958626436	50S ribosomal protein L20	1.3
212	4.4	gi|958626140	ArsR family transcriptional regulator	1.1
213	4.4	gi|944303984	aspartate aminotransferase	1.3
214	4.3	gi|595879770	thymidine phosphorylase	1.8
215	4.3	gi|944304869	iron transporter FeoA	1.7
216	4.3	gi|958622282	50S ribosomal protein L24	1.1
217	4.2	gi|942010853	valine--tRNA ligase	0.3
218	4.2	gi|958622292	adenylate kinase	0.4
219	4.2	gi|958623564	cytochrome b6	1.0
220	4.1	gi|958625649	2-oxoglutarate ferredoxin oxidoreductase subunit alpha	0.9
221	4.1	gi|940631484	6,7-dimethyl-8-ribityllumazine synthase	1.1
222	4.1	gi|958627256	single-stranded DNA-binding protein	0.9
223	4.1	gi|595878250	phage-shock protein	1.4
224	4.1	gi|310898922	unnamed protein product	0.3
225	4.1	gi|958626838	glycine cleavage system protein H	1.3
226	4.1	gi|958627122	50S ribosomal protein L31 type B	1.4
227	4.1	gi|942010397	oxidoreductase	0.8
228	4.1	gi|958626503	acetate kinase	0.9
229	4.0	gi|958624749	peptide ABC transporter substrate-binding protein	0.1

230	4.0	gi|942012354	phosphomethylpyrimidine kinase	1.1
231	4.0	gi|958626852	acetyl-CoA acetyltransferase	5.4
232	4.0	gi|942009894	glyoxalase	1.1
233	4.0	gi|942012137	PTS N-acetylglucosamine transporter subunit IIB	1.3
234	4.0	gi|942010442	purine nucleoside phosphorylase	0.9
235	4.0	gi|958622920	L-cystine-binding protein TcyA	0.5
236	4.0	gi|940632357	hypothetical protein AL712_11985	0.1
237	3.9	gi|958622274	50S ribosomal protein L2	0.9
238	3.9	gi|958626420	asparaginyl- tRNA synthetase	1.0
239	3.9	gi|958624250	methylisocitrate lyase	6.0
240	3.9	gi|944302212	Zn-dependent hydrolase	2.2
241	3.8	gi|958623233	peptide ABC transporter ATP-binding protein	1.0
242	3.8	gi|958627102	ATP F0F1 synthase subunit B	0.9
243	3.7	gi|958626725	2-ketocyclohexanecarboxyl-CoA hydrolase	0.7
244	3.7	gi|940629427	septation protein spoVG	1.2
245	3.7	gi|944302467	enoyl-CoA hydratase	
246	3.7	gi|958625760	aminopeptidase	0.7
247	3.6	gi|944302749	4-hydroxy-tetrahydrodipicolinate synthase	0.8
248	3.6	gi|958624205	acetylornithine deacetylase	0.7
249	3.6	gi|958627153	arginyl-tRNA synthetase	0.5
250	3.6	gi|958626343	Ohr subfamily peroxiredoxin	6.6
251	3.6	gi|944302741	inorganic pyrophosphatase	1.6
252	3.5	gi|940631911	acetyl-CoA synthetase	5.8

253	3.5	gi|940629530	superoxide dismutase	0.8
254	3.5	gi|958624348	Tellurite resistance protein	0.4
255	3.5	gi|595875831	cell division protein FtsH	0.5
256	3.4	gi|958626067	hypothetical protein ATN06_21100	1.0
257	3.4	gi|942010305	hypothetical protein BTXL6_02210	
258	3.4	gi|958625869	hypothetical protein ATN06_20040	1.6
259	3.4	gi|958625648	2-oxoacid ferredoxin oxidoreductase	1.2
260	3.3	gi|944304075	hypothetical protein AQ980_22535	1.0
261	3.3	gi|595875820	lysyl-tRNA synthetase	0.6
262	3.3	gi|958622533	chemical-damaging agent resistance protein C	0.9
263	3.3	gi|958623232	peptide ABC transporter ATP-binding protein	0.7
264	3.3	gi|942010725	30S ribosomal protein S20	1.0
265	3.2	gi|940631503	phosphopentomutase	0.7
266	3.2	gi|958625690	30S ribosomal protein S15	1.1
267	3.2	gi|958622273	50S ribosomal protein L23	1.3
268	3.2	gi|944304331	oligopeptidase PepB	1.0
269	3.2	gi|944303790	aspartate--ammonia ligase	5.9
270	3.2	gi|958625909	hypothetical protein ATN06_20250	1.3
271	3.1	gi|958622313	arginase	0.9
272	3.1	gi|940632066	stress protein	1.0
273	3.0	gi|958625689	polyribonucleotide nucleotidyltransferase	0.8
274	3.0	gi|958627134	3-hydroxybutyryl-CoA dehydrogenase	1.7
275	2.9	gi|958622764	cytochrome ubiquinol oxidase subunit I	0.8
276	2.9	gi|958625933	phosphocarrier protein HPr	1.0

277	2.9	gi|958623181	cold-shock protein	1.6
278	2.8	gi|958622304	30S ribosomal protein S9	1.0
279	2.8	gi|595880444	aspartate kinase	0.8
280	2.8	gi|595878261	dihydropteridine reductase	0.7
281	2.8	gi|944304540	peptidylprolyl isomerase	1.0
282	2.8	gi|940628822	CTP synthetase	0.8
283	2.7	gi|958626337	delta-aminolevulinic acid dehydratase	1.2
284	2.7	gi|940630206	GMP synthetase	
285	2.7	gi|940632174	septum site-determining protein MinD	0.9
286	2.6	gi|958627175	phosphotransacetylase	0.6
287	2.5	gi|944304854	glycine oxidase	1.8
288	2.5	gi|595880235	dipeptidase PepV	0.6
289	2.5	gi|958626662	S-ribosylhomocysteinase	1.0
290	2.4	gi|942010254	isoleucine--tRNA ligase	1.7
291	2.4	gi|958625712	ATP-dependent protease subunit HslV	1.3
292	2.4	gi|944304190	oligoendopeptidase F	0.9
293	2.4	gi|958625824	cytochrome ubiquinol oxidase subunit I	1.4
294	2.4	gi|942010012	imidazolonepropionase	3.3
295	2.3	gi|958622287	50S ribosomal protein L18	1.6
296	2.3	gi|958625791	cell division protein FtsZ	1.2
297	2.3	gi|944305398	peptide chain release factor 1	
298	2.3	gi|944305153	GTP-binding protein	
299	2.3	gi|944304570	hypothetical protein AQ980_25095	0.9
300	2.3	gi|942011254	hypothetical protein BTXL6_07180	0.7
301	2.3	gi|942010988	ATP-dependent 6-phosphofructokinase	2.1
302	2.2	gi|940631801	zinc protease	0.9

303	2.2	gi|958627428	cold-shock protein	0.2
304	2.2	gi|958627255	30S ribosomal protein S18	1.3
305	2.2	gi|958622280	30S ribosomal protein S17	1.0
306	2.2	gi|944303559	peptidase	0.2
307	2.2	gi|958622294	translation initiation factor IF-1	0.6
308	2.1	gi|940629227	serine--tRNA ligase	2.1
309	2.1	gi|958623469	sulfate adenylyltransferase	1.5
310	2.1	gi|940631953	metal-dependent hydrolase	2.3
311	2.1	gi|958623967	spore protein	1.1
312	2.1	gi|958623177	malate synthase	2.4
313	2.1	gi|942013796	acyl-CoA dehydrogenase	0.4
314	2.1	gi|942010856	glutamate-1-semialdehyde aminotransferase	2.5
315	2.1	gi|942010799	aspartate--tRNA ligase	1.2
316	2.1	gi|958622455	glutamyl-tRNA amidotransferase	0.7
317	2.1	gi|958622279	50S ribosomal protein L29	1.0
318	2.0	gi|944304174	ribonucleotide-diphosphate reductase subunit alpha	0.7
319	2.0	gi|944305208	ABC transporter substrate-binding protein	0.3
320	2.0	gi|958623852	2-dehydropantoate 2-reductase	0.6
321	2.0	gi|958626826	Fe-S cluster assembly protein SufB	1.0
322	2.0	gi|942013151	aminotransferase	2.9
323	2.0	gi|958623596	cell division protein GpsB	0.9
324	2.0	gi|940632705	aminoacyl-histidine dipeptidase	2.3
325	2.0	gi|595879687	chemotaxis protein CheY	1.8
326	2.0	gi|944304457	isocitrate lyase	1.8

327	2.0	gi|940628777	phosphoglucosamine mutase	1.4
328	2.0	gi|958626777	ferredoxin-NADP reductase	4.1
329	2.0	gi|958627111	ribose-5-phosphate isomerase	1.2
330	2.0	gi|958627179	spore coat protein GerQ	0.4
331	2.0	gi|944305312	NAD-dependent succinate-semialdehyde dehydrogenase	
332	2.0	gi|942012133	hypothetical protein BTXL6_12230	0.2
333	2.0	gi|940631292	elongation factor Tu	
334	2.0	gi|942010492	serine dehydratase	2.5
335	2.0	gi|958625754	guanylate kinase	
336	2.0	gi|958623669	two-component system response regulator	0.4
337	2.0	gi|942010795	Rrf2 family transcriptional regulator	
338	2.0	gi|958626363	helix-turn-helix transcriptional regulator	0.2
339	2.0	gi|558693272	short-chain dehydrogenase/reductase	
340	2.0	gi|958622685	UDP-glucose 4-epimerase	1.8
341	2.0	gi|958625732	acyl carrier protein	1.5
342	2.0	gi|958623486	hypothetical protein ATN06_07295	
343	2.0	gi|958623471	ferredoxin--nitrite reductase	
344	2.0	gi|958627097	ATP synthase F0F1 subunit epsilon	
345	2.0	gi|958623524	ferredoxin	
346	2.0	gi|944304468	AraC family transcriptional regulator	1.7
347	2.0	gi|958622676	ArsR family transcriptional regulator	1.0

Supplementary Table S5. Summary of the identified proteins by iTRAQ searched by the whole *B. thuringiensis* database including all subspecies in NCBI.

	Unused	Accession	Gene	Protein names	Length	**117:114** (treatment sample: control sample)
1	3.0	sp|Q633M1|IF 3_BACCZ	infC	Translation initiation factor IF-3	167	5.4
2	7.6	sp|Q6HPP4|RS 8_BACHK	rpsH	30S ribosomal protein S8	132	5.1
3	13.5	sp|Q6HBD8|C LPP2_BACH K	clpP2	ATP-dependent Clp protease proteolytic subunit 2	193	4.2
4	7.5	sp|Q6HPP0|R L30_BACHK	rpmD	50S ribosomal protein L30	60	4.0
5	2.1	sp|Q6HBX6|F ENR2_BACH K	BT9727 _4639	Ferredoxin--NADP reductase 2	331	3.6
6	38.5	sp|Q6HEX9|S UCC_BACHK	sucC	Succinyl-CoA ligase [ADP-forming] subunit beta	386	3.5
7	2.8	sp|Q6HAX6|A TPD_BACHK	atpH	ATP synthase subunit delta	180	3.4
8	40.1	sp|Q6HPC7|C H60_BACHK	groL	60 kDa chaperonin	544	3.2
9	25.3	sp|Q6HJ19|IO LA1_BACHK	iolA1	Methylmalonate semialdehyde dehydrogenase [acylating] 1	486	3.1
10	2.5	sp|A0RH37|H UTI_BACAH	hutI	Imidazolonepropionase	423	2.9
11	8.9	sp|Q6HPS1|R L11_BACHK	rplK	50S ribosomal protein L11	141	2.9
12	6.3	sp|Q6HLV6|Y	BT9727	Putative phosphoesterase	172	2.8

		1129_BACHK	_1129			
13	2.2	sp|Q6HPZ8|SYS_BACHK	serS	Serine--tRNA ligase	424	2.7
14	5.6	sp|A0RFE8|MQO_BACAH	mqo	Probable malate:quinone oxidoreductase	500	2.6
15	16.1	sp|Q6HJK9|FTHS_BACHK	fhs	Formate--tetrahydrofolate ligase	562	2.5
16	16.9	sp|Q6HPQ2|RS3_BACHK	rpsC	30S ribosomal protein S3	219	2.4
17	12.5	sp|Q6HPR2|RS7_BACHK	rpsG	30S ribosomal protein S7	156	2.3
18	53.7	sp|Q6HP91|ROCA_BACHK	rocA	1-pyrroline-5-carboxylate dehydrogenase	515	2.0
19	10.2	sp|Q637H9|HUTU_BACCZ	hutU	Urocanate hydratase	552	1.9
20	6.1	sp|Q6HBZ1|SYG_BACHK	glyQS	Glycine--tRNA ligase	458	1.9
21	2.3	sp|Q6HCT3|PFKA_BACHK	pfkA	ATP-dependent 6-phosphofructokinase	319	1.8
22	2.3	sp|Q6HER9|SYI1_BACHK	ileS1	Isoleucine--tRNA ligase 1	921	1.8
23	11.6	sp|Q6HPQ8|RL3_BACHK	rplC	50S ribosomal protein L3	210	1.7
24	21.1	sp|Q6HAW9|GLYA_BACHK	glyA	Serine hydroxymethyltransferase	414	1.7
25	14.4	sp|Q6HN84|THIG_BACHK	thiG	Thiazole synthase	258	1.7
26	7.4	sp|Q6HEX1|RS16_BACHK	rpsP	30S ribosomal protein S16	90	1.6
27	4.0	sp|Q6HAG2|RS6_BACHK	rpsF	30S ribosomal protein S6	96	1.6

28	19.5	sp|Q6HPP6|RL5_BACHK	rplE	50S ribosomal protein L5	179	1.5
29	33.3	sp|Q6HBF3|ENO_BACHK	eno	Enolase	431	1.4
30	3.7	sp|Q6HE53|RISB_BACHK	ribH	6,7-dimethyl-8-ribityllumazine synthase	153	1.4
31	3.6	sp|Q6HPC8|CH10_BACHK	groS	10 kDa chaperonin	94	1.3
32	2.0	sp|Q6HEW5|ACP_BACHK	acpP	Acyl carrier protein	77	1.3
33	9.1	sp|Q6HC14|LDH2_BACHK	ldh2	L-lactate dehydrogenase 2	314	1.3
34	4.5	sp|Q6HDM4|PDRP_BACHK	BT9727_4034	Putative pyruvate, phosphate dikinase regulatory protein	270	1.3
35	9.8	sp|Q6HD54|CLPX_BACHK	clpX	ATP-dependent Clp protease ATP-binding subunit ClpX	419	1.3
36	6.8	sp|Q6HAP7|Y5069_BACHK	BT9727_5069	Putative heme-dependent peroxidase	247	1.3
37	3.6	sp|Q6HHR6|PPAC_BACHK	ppaC	Probable manganese-dependent inorganic pyrophosphatase	309	1.3
38	7.2	sp|Q6HPN9|RL15_BACHK	rplO	50S ribosomal protein L15	146	1.2
39	14.2	sp|Q6HPP1|RS5_BACHK	rpsE	30S ribosomal protein S5	166	1.2
40	4.2	sp|Q6HAV2|RL31B_BACHK	rpmE2	50S ribosomal protein L31 type B	81	1.2
41	21.0	sp|Q634V7|GCSPA_BACCZ	gcvPA	Probable glycine dehydrogenase	447	1.2

42	10.5	sp|Q6HD81|RL21_BACHK	rplU	50S ribosomal protein L21	102	1.2
43	2.1	sp|A0R8M4|GLMM_BACAH	glmM	Phosphoglucosamine mutase	448	1.2
44	10.1	sp|Q6HBQ8|LDH3_BACHK	ldh3	L-lactate dehydrogenase 3	316	1.2
45	5.2	sp|Q6HCV7|RL20_BACHK	rplT	50S ribosomal protein L20	118	1.2
46	3.8	sp|Q6HD68|SYV_BACHK	valS	Valine--tRNA ligase	881	1.2
47	2.0	sp|Q6HKD8|ASNA_BACHK	asnA	Aspartate--ammonia ligase	327	1.2
48	9.4	sp|Q6HPP3|RL6_BACHK	rplF	50S ribosomal protein L6	179	1.1
49	7.8	sp|Q6HPM6|RL13_BACHK	rplM	50S ribosomal protein L13	145	1.1
50	3.1	sp|Q6HPQ6|RL23_BACHK	rplW	50S ribosomal protein L23	96	1.1
51	2.1	sp|Q6HEU4|KGUA_BACHK	gmk	Guanylate kinase	205	1.1
52	5.7	sp|Q6HDF1|MTNN_BACHK	mtnN	5'-methylthioadenosine/S-adenosylhomocysteine nucleosidase	231	1.1
53	22.7	sp|Q632S4|PCKA_BACCZ	pckA	Phosphoenolpyruvate carboxykinase [ATP]	528	1.1
54	8.7	sp|Q6HPN1|RL17_BACHK	rplQ	50S ribosomal protein L17	120	1.1
55	4.3	sp|Q6HPW9|SP5G_BACHK	spoVG	Putative septation protein SpoVG	97	1.1
56	7.7	sp|Q6HEI4|D	dapH	2,3,4,5-tetrahydropyridine-2,6-	240	1.1

		APH_BACHK		dicarboxylate N-acetyltransferase		
57	10.6	sp|Q6HEY7|CODY_BACHK	codY	GTP-sensing transcriptional pleiotropic repressor CodY	259	1.1
58	24.8	sp|Q6HAX7|ATPA_BACHK	atpA	ATP synthase subunit alpha	502	1.1
59	4.2	sp|Q6HBR6|GCSH_BACHK	gcvH	Glycine cleavage system H protein	127	1.1
60	13.7	sp|Q6HPR9|RL10_BACHK	rplJ	50S ribosomal protein L10	166	1.0
61	11.7	sp|Q6HCM2|RS4_BACHK	rpsD	30S ribosomal protein S4	200	1.0
62	8.1	sp|Q6HMV9|Y768_BACHK	BT9727_0768	UPF0342 protein BT9727_0768	118	1.0
63	9.3	sp|Q6HPQ3|RL22_BACHK	rplV	50S ribosomal protein L22	113	1.0
64	6.4	sp|Q6HEX5|RL19_BACHK	rplS	50S ribosomal protein L19	114	1.0
65	5.8	sp|Q6HDY4|FOLD_BACHK	folD	Bifunctional protein FolD	286	1.0
66	5.7	sp|P23382|INA_BACTL	ina	Immune inhibitor A	687	1.0
67	2.1	sp|Q634E3|SYDND_BACCZ	aspS	Aspartate--tRNA(Asp/Asn) ligase	591	1.0
68	21.8	sp|Q6HPR6|RPOB_BACHK	rpoB	DNA-directed RNA polymerase subunit beta	1177	1.0
69	4.5	sp|Q6HPP7|RL24_BACHK	rplX	50S ribosomal protein L24	103	1.0
70	3.7	sp|Q6HPQ7|RL4_BACHK	rplD	50S ribosomal protein L4	207	1.0
71	2.6	sp|Q6HAG4|R	rpsR	30S ribosomal protein	77	1.0

		S18_BACHK		S18		
72	34.3	sp|Q6HAY0|ATPB_BACHK	atpD	ATP synthase subunit beta	469	1.0
73	2.4	sp|Q6HPP9|RS17_BACHK	rpsQ	30S ribosomal protein S17	87	1.0
74	4.4	sp|Q6HCN5|NADK2_BACHK	nadK2	NAD kinase 2	267	1.0
75	4.0	sp|Q6HF07|RS15_BACHK	rpsO	30S ribosomal protein S15	89	1.0
76	20.4	sp|Q6HDK7|DNAK_BACHK	dnaK	Chaperone protein DnaK	611	1.0
77	17.9	sp|Q63H97|RPOC_BACCZ	rpoC	DNA-directed RNA polymerase subunit beta'	1203	1.0
78	17.4	sp|Q6HDT8|GCSPB_BACHK	gcvPB	Probable glycine dehydrogenase (decarboxylating) subunit 2	491	1.0
79	10.1	sp|Q6HL40|NDK_BACHK	ndk	Nucleoside diphosphate kinase	148	1.0
80	2.1	sp|P0A4I4|SP0A_BACTU	spo0A	Stage 0 sporulation protein A	264	1.0
81	8.3	sp|A0RJZ7|METK_BACAH	metK	S-adenosylmethionine synthase	399	1.0
82	13.7	sp|Q6HEY8|RS2_BACHK	rpsB	30S ribosomal protein S2	233	0.9
83	7.8	sp|Q6HPQ9|RS10_BACHK	rpsJ	30S ribosomal protein S10	102	0.9
84	8.7	sp|Q6HAX9|ATPG_BACHK	atpG	ATP synthase gamma chain	286	0.9
85	6.0	sp|Q6HPP8|RL14_BACHK	rplN	50S ribosomal protein L14	122	0.9

86	3.7	sp|Q6HCW9|SYN_BACHK	asnS	Asparagine--tRNA ligase	463	0.9
87	2.8	sp|Q6HDJ9|RS20_BACHK	rpsT	30S ribosomal protein S20	85	0.9
88	6.2	sp|Q6HD83|RL27_BACHK	rpmA	50S ribosomal protein L27	96	0.9
89	2.8	sp|Q6HPM5|RS9_BACHK	rpsI	30S ribosomal protein S9	130	0.9
90	2.5	sp|Q6HC89|LUXS_BACHK	luxS	S-ribosylhomocysteine lyase	157	0.9
91	2.1	sp|Q6HD65|GSA2_BACHK	hemL2	Glutamate-1-semialdehyde 2,1-aminomutase 2	429	0.9
92	14.3	sp|Q6HPS0|RL1_BACHK	rplA	50S ribosomal protein L1	230	0.9
93	5.5	sp|Q6HPN4|RS13_BACHK	rpsM	30S ribosomal protein S13	121	0.9
94	4.5	sp|Q6HAX5|ATPF_BACHK	atpF	ATP synthase subunit b	168	0.9
95	10.1	sp|Q6HD53|TIG_BACHK	tig	Trigger factor	425	0.9
96	2.5	sp|Q9F166|PTHP_BACTI	ptsH	Phosphocarrier protein HPr	87	0.9
97	12.7	sp|Q6HF02|IF2_BACHK	infB	Translation initiation factor IF-2	686	0.9
98	12.4	sp|Q6HBF1|TPIS_BACHK	tpiA	Triosephosphate isomerase	251	0.9
99	4.9	sp|Q6HC08|G6PI_BACHK	pgi	Glucose-6-phosphate isomerase	450	0.9
100	12.2	sp|Q6HCU0|MDH_BACHK	mdh	Malate dehydrogenase	312	0.8
101	8.1	sp|Q6HPR8|RL7_BACHK	rplL	50S ribosomal protein L7/L12	119	0.8

102	7.3	sp|Q6HPQ4|RS19_BACHK	rpsS	30S ribosomal protein S19	92	0.8
103	7.0	sp|Q6HEZ1|RRF_BACHK	frr	Ribosome-recycling factor	185	0.8
104	6.1	sp|Q6HPN2|RPOA_BACHK	rpoA	DNA-directed RNA polymerase subunit alpha	314	0.8
105	5.2	sp|Q6HMD5|GLPK_BACHK	glpK	Glycerol kinase	496	0.8
106	4.4	sp|Q6HPN3|RS11_BACHK	rpsK	30S ribosomal protein S11	129	0.8
107	4.2	sp|Q633F8|ACKA_BACCZ	ackA	Acetate kinase	397	0.8
108	2.1	sp|Q6HL37|HIS81_BACHK	hisC1	Histidinol-phosphate aminotransferase 1	370	0.8
109	5.1	sp|Q6HBY2|AMPA_BACHK	pepA	Probable cytosol aminopeptidase	494	0.8
110	2.5	sp|Q6HMG1|Y921_BACHK	BT9727_0921	UPF0145 protein	103	0.8
111	2.0	sp|Q6HPQ1|RL16_BACHK	rplP	50S ribosomal protein L16	144	0.8
112	6.4	sp|Q6HPN7|KAD_BACHK	adk	Adenylate kinase	216	0.8
113	2.9	sp|Q6HF08|PNP_BACHK	pnp	Polyribonucleotide nucleotidyltransferase	712	0.7
114	9.1	sp|A0RI12|METE_BACAH	metE	5-methyltetrahydropteroyltriglutamate--homocysteine methyltransferase	762	0.7
115	4.7	sp|Q6HQ05|PDXS_BACHK	pdxS	Pyridoxal 5'-phosphate synthase subunit PdxS	295	0.7
116	3.6	sp|Q6HPQ5|R	rplB	50S ribosomal protein L2	276	0.7

		L2_BACHK				
117	10.2	sp|Q6HPL2|GLMS_BACHK	glmS	Glutamine--fructose-6-phosphate aminotransferase [isomerizing]	600	0.7
118	2.1	sp|Q63GQ9|GATB_BACCZ	gatB	Aspartyl/glutamyl-tRNA(Asn/Gln) amidotransferase subunit B	475	0.7
119	3.0	sp|Q6HE79|DEOB_BACHK	deoB	Phosphopentomutase	394	0.6
120	21.4	sp|Q6HEY6|HSLU_BACHK	hslU	ATP-dependent protease ATPase subunit HslU	463	0.6
121	19.4	sp|Q6HDT6|GCST_BACHK	gcvT	Aminomethyltransferase	366	0.6
122	2.3	sp|Q6HEP3|Y3663_BACHK	BT9727_3663	Uncharacterized N-acetyltransferase	157	0.5
123	8.2	sp|Q6HBD4|Y4833_BACHK	BT9727_4833	Nucleotide-binding protein	293	0.5
124	2.3	sp|Q6HPN5|IF1_BACHK	infA	Translation initiation factor IF-1	72	0.5
125	21.1	sp|Q6HEY9|EFTS_BACHK	tsf	Elongation factor Ts	295	0.5
126	3.0	sp|Q6HAS1|SYR2_BACHK	argS2	Arginine--tRNA ligase 2	556	0.5
127	4.8	sp|Q6HNE4|TAL_BACHK	tal	Probable transaldolase	215	0.4
128	15.1	sp|Q631L9|PGK_BACCZ	pgk	Phosphoglycerate kinase	394	0.4
129	10.5	sp|Q6HP28|OTCC_BACHK	arcB	Ornithine carbamoyltransferase, catabolic	332	0.4
130	34.1	sp|Q6HPR0|E	tuf	Elongation factor Tu	395	0.3

		FTU_BACHK		(EF-Tu)		
131	29.6	sp|Q6HP29|ARCA_BACHK	arcA	Arginine deiminase	410	0.2
132	2.6	sp|Q6HLA6|HMP_BACHK	hmp	Flavohemoprotein	402	0.2
133	5.7	sp|Q63GV4|GUAA_BACCZ	guaA	GMP synthase [glutamine-hydrolyzing]	515	0.2
134	42.6	sp|Q63H93|EFG_BACCZ	fusA	Elongation factor G (EF-G)	692	
135	2.4	sp|Q6HAV4|RF1_BACHK	prfA	Peptide chain release factor 1	355	
136	2.1	sp|Q633I4|Y4354_BACCZ	BCE33L4354	UPF0173 metal-dependent hydrolase	227	
137	2.0	sp|Q6HPR1|EFG_BACHK	fusA	Elongation factor G (EF-G)	692	
138	2.0	sp|Q6HAY1|ATPE_BACHK	atpC	ATP synthase epsilon chain	133	

Supplementary Table S6. Summary of the identified proteins by iTRAQ searched by the *B. thuringiensis* database in UNIPROT (unused scores>2).

Number	protein	Protein website	Possible binding site number	Pose number of binding sites*
1	acetyl-CoA acetyltransferase (gi|118418455)	Swiss modle	10	site 1: 83; site 3: 100; site 4: 100; site 6: 100; site 7: 100; site 8: 100; site 9: 95
		Phyre^2	6	site 1: 41
2	alkyl hydroperoxide reductase C22 (gi|326938119) **	Swiss modle	9	site 1: 100; site 5: 1
		Phyre^2	5	
3	SOD (gi|449026274)	Swiss modle	12	site 1: 8; site 2: 6; site 3: 21; site 7: 98
		Phyre^2	7	site 1: 100
4	leucine dehydrogenase (gi|449024882)	Swiss modle	12	site 1: 100; site 3: 100; site 4: 100
		Phyre^2	12	site 1: 100; site 2: 37; site 4: 5; site 9: 1
5	electron transfer flavoprotein subunit alpha (gi|401792614)	Swiss modle	11	site 1: 100; site 2: 10; site 5: 26; site 7: 4; site 8: 22
		Phyre^2	10	site 1: 6; site 3: 9; site 4: 100; site 5: 12; site 6: 100
6	branched-chain alpha-keto acid dehydrogenase E1 component (gi|118418579)	Swiss modle	12	site 1: 5; site 3: 1; site 4: 3
		Phyre^2	15	site 1: 3; site 2: 60; site 3: 13
7	phosphoenolpyruvate carboxykinase (gi|296326283)	Swiss modle	10	site 1: 100; site 2: 100; site 4: 7; site 5: 100; site 6: 100; site 8: 100
		Phyre^2	10	site 1: 100; site 2: 4; site 3: 3; site 7: 100; site 9: 8

Note:

[*]: Pose number of binding sites without showing above is zero.

[**]: Only A and B chains of alkyl hydroperoxide reductase C22 from Swiss modle were analyzed, because other chains were the same with A and B chains.

Supplementary Table S7. Possible binding site number of proteins and pose number of TPT.

Methods for 2DE and iTRAQ

Protein preparation. Cells before and after TPT treatment were suspended in 1 mL lysis buffer containing 15 mM Tris-HCl, 7 M urea, 2 M thiourea, 1% w/v dithiothreitol (DTT) and 4% w/v 3-[(3-cholamidopropyl)dimethylammonio]propanesulfonate (CHAPS), and then added with 0.2 g L^{-1} phenylmethylsulfonyl fluoride, 2% v/v IPG buffer and 0.6 g L^{-1} DTT. After vortexing, the samples were frozen in liquid nitrogen 3 times (15 min per time) and subsequently treated by ultrasonication for 20 min. A nuclease mix at a final concentration of 1% v/v was added to the lysate and incubated at 4 ℃ for 30 min. The protein concentration after centrifugation was measured using the Bradford method. Proteins from untreated cells were mixed with 1 mg L^{-1} TPT or without TPT to determine the interaction between protein primary structure and TPT. In addition, protein samples from cells with TPT treatment for 1 d and control samples were prepared for iTRAQ.

Two-dimensional gel electrophoresis and image generation. The above protein samples without TPT treatment were diluted with hydration solution (7 M urea, 2 M thiourea, 2% w/v CHAPS) to the concentration of 0.4 g L^{-1} to which 0.3 g L^{-1} DTT, 0.5% v/v IPG buffer and 0.02‰ w/v bromophenol blue were added. A 2DE was run for each sample of 100 μg purified

whole protein in 250 μL solution. The first dimension electrophoresis for protein separation was conducted in isoelectric focusing (IEF) IPG-strip gels (13 cm, pH 4–7, GE Healthcare) at 20 ℃ with Ettan IPGphor system (GE Healthcare BIO-Science). Samples were first loaded by passive rehydration for 12 h. The IEF was run at 30 V for 12 h, 100 V for 1 h, 200 V for 1 h, 500 V for 1 h, 1000 V for 1 h, followed by a linear increase to 8000 V where it was held until 32000 Vhr was obtained. After IEF, the strips were soaked first in equilibration buffer (6 M urea, 2% w/v sodium dodecyl sulfate (SDS), and 50 mM Tris-HCl, pH 8.8, 30% w/v glycerol and 0.02‰ w/v bromophenol blue) containing 1% w/v DTT for 15 min and then in the equilibration buffer with 2.5% w/v iodoacetamide for 15 min. The equilibrated strips were inserted on the top of a 12.5% SDS separating polyacrylamide gel and were covered with 0.5% w/v low melting point agarose. TPT at 1 mg L^{-1} was added in low melting point agarose and used to interact with proteins in the IPG-strip gels during the electrophoresis process. The result was compared with control sample without TPT treatment and was used to verify interactions between TPT and protein primary structure. The gels were run in a polyacrylamide gel electrophoresis system (Hoefer SE600, GE Healthcare) at a constant current of 5 mA gel^{-1} for 30 min and then run at 15 mA gel^{-1} until the marker dye reached the bottom of the gels.

The gels were stained with silver nitrate following electrophoresis. Electrophoretic gels were fixed using a solution of 40% ethyl alcohol and 10% acetic acid for 12 h and sensitized in sensitizing solution (0.2% w/v sodium thiosulfate, 30% ethyl alcohol and 6.8% w/v sodium acetate) for 30 min. To perform silver staining, gels were soaked for 30 min in 0.25% w/v silver nitrate and 0.02% v/v formaldehyde. The gels were then washed four times for 10 min in pure water. Subsequently, silver-stained gels were reduced by soaking in 2.5 g L^{-1} sodium carbonate containing 0.2‰ v/v formaldehyde for 5 min. Finally, the staining was stopped by 10 min incubation in 1.46% w/v EDTA–Na $\cdot 2H_2O$. In addition, silver nitrate staining solution with 1 mg L^{-1} TPT added was used to stain the effector proteins in polyacrylamide gels to evaluate the interaction between TPT and protein primary structure.

Later, the gels were scanned by the ImageScanner II (GE Healthcare) and analyzed with the Progenesis Samespots software. Proteins were considered differentially expressed when the average values exceeded the two-fold threshold with a p-value<0.05.

In-gel digestion and protein identification. Silver-stained protein spots of interest were cut out from the gels and destained in 50 μL of 15 mM potassium ferricyanide and 50 mM sodium thiosulfate. The gel pieces were serially dehydrated with 50 μL 50% acetonitrile and 50 μL 100% acetonitrile and then rehydrated with 5 μL of 25 mM ammonium bicarbonate containing

13 ng of trypsin (Promega, V5280, USA) at 4 ℃ for 30 min. The in-gel proteins were digested by incubating at 37 ℃ for 18 h. The digestion was terminated with trifluoroacetic acid at a final concentration of 2.5% v/v. The peptides were dried in a vacuum concentrator, and then resolved with 1.2 μL solution (30% v/v acetonitrile, 0.1% v/v trifluoroacetic acid). One microliter of the above sample was deposited on a SCOUTMTPMALDI target chip, and 0.5 μL of 5 g L^{-1} a-cyano-4-hydroxycinnamic acid was added and subsequently analyzed by an ABI 4800 MALDI TOF/TOF mass spectrometer (Applied Biosystems, Foster City, CA; protein score>95%).

In-solution trypsin digestion for iTRAQ analysis. Proteins from cells with or without TPT treatment for 1 d were reduced with 2 μL reducing reagent in the iTRAQ reagent multiplex kit (Sigma, PN 4352135, USA) for 1 h at 37 ℃. Cysteines were blocked with 1 μL blocking reagent provided in the kit for 10 min. The protein samples were added to 10 KD Amicon Ultra-0.5 centrifugal filter devices, centrifuged at 12000 r min^{-1} for 20 min, and washed three times with 100 μL dissolution buffer. After removing the liquid in collection tube, the samples in filter devices were digested by 50 μL trypsin (Promega, V5280, USA) at 4% W/W overnight at 37 ℃. Subsequently, the samples were centrifuged, and 1 μg trypsin was added to each filter for 2 h. The liquid in collection tubes was collected after centrifugation and measured using the Bradford method.

iTRAQ labeling, desalination and protein identification. Tryptic peptides were labeled with an iTRAQ reagent multiplex kit (Reagent 114: ^{18}O content is 91–97%; Reagent 117: ^{15}N content is 98–100%) according to the instructions. In total, 150 μL ethanol was added to each tube of iTRAQ reagent and vortexed. Reagents 114 and 117 were used to label proteins extracted from the control samples and the treated samples, respectively. Therefore, 117:114 means the protein level of the samples after TPT treatment compared with the control samples.

After transferring the tryptic peptides to a new tube, the above solution was added into the tube and incubated for 2 h. In total, 100 μL water was added to each sample to stop the reaction. One microliter of solution from each sample was detected by ABI 4800 MALDI TOF/TOF (Applied Biosystems, Foster City, CA) to determine the labeling efficiency. Subsequently, the iTRAQ-labeled samples were mixed, vortexed, centrifuged, desalinated with Strata-X (Phenomenex, USA), and separated by strong cation exchange chromatography. The labeled peptides were dried in a vacuum concentrator. The samples were then dissolved in solution (2% v/v acetonitrile, 0.1% v/v formic acid), centrifuged at 12000 r min^{-1} 3 times (20 min each time), and analyzed by an AB Sciex TripleTOF 5600 mass spectrometer (AB Sciex, Framingham, MA, USA) equipped with a Nanospray III source (AB Sciex). The unused scores of all the proteins

for further study were above 2. 117:114 scores of 1.2 or .8333 were used as thresholds for significant up- or downregulation, respectively.
